# *Asic3^−/−^* Female Mice with Hearing Deficit Affects Social Development of Pups

**DOI:** 10.1371/journal.pone.0006508

**Published:** 2009-08-04

**Authors:** Wei-Li Wu, Chih-Hung Wang, Eagle Yi-Kung Huang, Chih-Cheng Chen

**Affiliations:** 1 Graduate Institute of Life Sciences, National Defense Medical Center, Taipei, Taiwan, Republic of China; 2 Institute of Biomedical Sciences, Academia Sinica, Nankang, Taipei, Taiwan, Republic of China; 3 Department of Otolaryngology-Head and Neck Surgery, Tri-Service General Hospital, Taipei, Taiwan, Republic of China; 4 Department of Pharmacology, National Defense Medical Center, Taipei, Taiwan, Republic of China; Centre National de la Recherche Scientifique, France

## Abstract

**Background:**

Infant crying is an important cue for mothers to respond adequately. Inappropriate response to infant crying can hinder social development in infants. In rodents, the pup-mother interaction largely depends on pup's calls. Mouse pups emit high frequency to ultrasonic vocalization (2–90 kHz) to communicate with their dam for maternal care. However, little is known about how the maternal response to infant crying or pup calls affects social development over the long term.

**Methodology/Principal Findings:**

Here we used mice lacking acid-sensing ion channel 3 (*Asic3^−/−^*) to create a hearing deficit to probe the effect of caregiver hearing on maternal care and adolescent social development. Female *Asic3^−/−^* mice showed elevated hearing thresholds for low to ultrasonic frequency (4–32 kHz) on auditory brain stem response, which thus hindered their response to their pups' wriggling calls and ultrasonic vocalization, as well as their retrieval of pups. In adolescence, pups reared by *Asic3^−/−^* mice showed a social deficit in juvenile social behaviors as compared with those reared by wild-type or heterozygous dams. The social-deficit phenotype in juvenile mice reared by *Asic3^−/−^* mice was associated with the reduced serotonin transmission of the brain. However, *Asic3^−/−^* pups cross-fostered to wild-type dams showed rescued social deficit.

**Conclusions/Significance:**

Inadequate response to pups' calls as a result of ASIC3-dependent hearing loss confers maternal deficits in caregivers and social development deficits in their young.

## Introduction

Hearing an infant's crying and responding to it is one of the most important ways to establish infant-mother interaction[1], [2]. Infants cry to let their mothers or caregivers know their discomfort: hunger, coldness, urination, pain, and desire to be held[2]. Maternal responsiveness to infant crying profoundly affects the infant-mother bonding, although two longitudinal studies concluded opposite effects[3], [4]. Nevertheless, social development could be hindered in infants with insecure attachment to their mothers. Children growing up with insecure attachment show reduced interaction with peers and low desire to explore an unfamiliar environment[5].

The mother's auditory functions play an important role in responding to infant crying. Mothers with deficits in auditory functions could ignore infant crying and fail to provide prompt response to their children, which may affect the infant-mother bonding[6]. However, little is known about how the acuity of the mother's hearing affects infant-mother bonding, and no longitudinal study has investigated the effect of the infant-mother attachment on social development in adolescence or adulthood.

In rodent studies, mouse pups receiving high levels of maternal care show increased neurotrophin levels in the brain, which promotes sociability in adulthood[7]. However, most research into mice maternal behaviors has focused on the effects of the pup's central nervous system and social development and not the caregiver's auditory sensation related to pup-mother attachment. In rodents, the pup-mother interaction largely depends on wriggling calls and ultrasonic vocalization (USV) ranging from 2–20 kHz and 30–90 kHz respectively[8]. Mouse pups emit wriggling calls to communicate with their dam, when they struggle in the nest[9]. The mother mouse responds to wriggling calls with three types of maternal behaviors: licking of pups, changes of suckling position, and nest building[10]. In addition, mouse pups emit USV when they are separated from their mother or are under uncomfortable situations[11], [12]. These distress calls can elicit the dam's approach and retrieval behaviors[13]. However, the molecular and neural basis of hearing sensation in response to wriggling calls and USV is largely unknown.

Acid sensing ion channels (ASICs) are voltage-independent sodium channels activated by external acidification[14], [15]. ASICs are expressed in the peripheral and central nervous systems, including the auditory system[16]–[18]. In the inner ear, both ASIC2 and ASIC3 are distributed in neurons of cochlear spiral ganglia, which transmit the hearing signaling from hair cells to the brain. Whole-cell patch recording studies showed that spiral ganglion neurons respond to protons and generate inward currents. This proton-induced response could be attenuated by amiloride, a nonspecific blocker for ASICs. The proton-induced response in spiral ganglion neurons is largely reduced in *Asic2^−/−^* mice, but ASIC2 seems to play a role in noise susceptibility rather than normal hearing ability[16]. In contrast, in studies of click-evoked auditory brainstem response (ABR), mice lacking *Asic3* showed progressive hearing loss with age[17]. However, *Asic3^−/−^* mice have never been investigated for hearing conditions at high frequencies or ultrasonic ranges. Since ASIC3 is predominantly expressed in sensory neurons but shows only low expression in the brain, we could use *Asic3^−/−^* mice to investigate the effect of hearing deficit on maternal behaviors without eliciting a central-nervous-system effect[19], [20].

In this study, we aimed to determine the effect of ASIC3-dependent hearing deficit on response to pup's calls, and maternal behaviors in mice. In addition, we conducted a longitudinal investigation of the *Asic3^−/−^* mouse model to explore how maternal response to pup's calls affects the social development of pups in adolescence.

## Materials and Methods

### Animals

The generation of *Asic3* knockout (*Asic3^−/−^*) mice was as described[21]. To dilute the effect of genetic background, the F_2_ *Asic3^−/−^* mice were backcrossed to CD-1 mice for at least 8 generations to generate outbred *Asic3^+/−^* mice. Mice used in this study were derived from the *Asic3^+/−^* mice intercross or offspring from the next generation of the *Asic3^−/−^* mice intercross. To average out the home cage effect, mice used in this study were derived from as many breeding pairs as possible. Whenever possible, littermates were used in an experimental set. CD-1 mice were chosen because they are good caregivers. Mice were group-housed (3–5) in a cage in a 12-h light/12-h dark cycle (lights on at 08:00 h) at 25 °C and 40–70% humidity. All experiments involved female mice 5–16 weeks old, juvenile male mice 5 weeks old, and postnatal pups 1–3 days old (P1-P3). The experimental protocol was approved by the Institutional Animal Care and Use Committee of Academia Sinica.

### ABR

Auditory functions were measured by evoked ABR. In brief, mice were anesthetized by intraperitoneal injection of sodium pentobarbital and kept warm with a heating pad in a sound-attenuating chamber. Subdermal needle electrodes were inserted at the vertex (positive), below the pinna of the ear (negative), and at the back (ground) of the mice. Specific stimuli (clicks and 4-, 8-, 16-, 32-kHz tone bursts) were generated by use of SigGen software (Tucker-Davis Technologies, Gainsville, FL) and delivered to the external auditory canal. The average responses from 1024 stimuli for each frequency were obtained by reducing the sound intensity in 5-dB steps until threshold. Thresholds were defined as the lowest intensity at which a reproducible deflection in the evoked response trace could be recognized.

### Pup retrieval tests

Virgin female mice 8∼16 weeks old were single-housed for 1 day before the experiment. Nesting material was provided in each cage for building a nest. The next day, three wild-type littermate pups (P1-P3) from *Asic3^+/+^* breeding pairs were isolated from their dam for 10 min in a novel cage with a heating lamp to keep the room temperature at 35°C and then placed in each of the three corners of the virgin mouse cage that did not contain a nest. The activity in the cage was videorecorded digitally for 30 min on this day and the next day. Activities on the first and second day were scored. The maternal behaviors scored were as follows:

#### Investigation latency

Time to investigate the first pup.

#### Pup retrieval latency

Time to retrieve each pup into the nest.

#### Duration of licking/grooming/sniffing pups

Time spent licking/grooming/sniffing pups, regardless of whether the pup was in the nest.

#### Crouching behaviour

When the virgin mouse used an arched-back posture to cover the pup in the nest. Crouching duration was the time mice spent crouching after all three pups were retrieved.

#### Pup injury

After retrieval test, the pups were carefully examined. The wounds on their bodies were recorded.

### USV recording during pup retrieval

The protocol was similar to the pup retrieval test, except only one pup was placed opposite the nest. A USV recorder (Anabat SD1 Bat Detector, Titley Elecronics, Ballina, NSW, Australia) was placed on the top of cage to record the USV of the pup for 10 min. At the same time, maternal behavior was videotaped from the side of the cage. Ten virgin mice in each genotype were involved in this study. A virgin mouse encountered 3 different wild-type littermate (P1∼P3) pups. USVs emitted by pups were discrete segments on the sonogram. We defined a segment of USVs as a bout. Parameters of behavior and USVs scored were as follows:

#### Calling rate

Number of vocalizations in a bout.

#### Average duration

Average duration of each vocalization pit in a bout.

#### Bout duration

Total time of a bout.

#### Approach latency

Time between the last call of a USV bout and when the virgin mouse sniffed the pup.

For each bout, maximal, minimal and mean sound frequency (Fmax, Fmin, Fmean, respectively) were characterized.

### Juvenile social behaviors

Postnatal 5-week-old mice (littermates) in group-housing were isolated for 3.5∼5 hours. After isolation, the tested mouse was placed in a new cage. Then a control wild-type mouse of similar age and size was immediately introduced into the cage. The social behaviors and USVs of the two mice were recorded for 15 min as described for the pup retrieval test. The affiliative behaviors scored were as follows:

#### Latency

Time for the tested (or introduced) mouse initiated the approach.

#### Anogenital sniffing

The time spent sniffing the partner's anogenital region.

#### Body sniffing

Time spent sniffing the partner's body region.

#### Whisker to whisker

The time tested mice spent on whisker contact with the partner mouse.

#### Mounting

The duration of approaching the partner and assuming a copulatory position.

#### Push under

Time spent drilling into partner's bottom side.

#### Touching

Time spent touching partner's body with a forepaw.

### Neurochemical measurement

Mice were immediately sacrificed after isolation (3.5∼5 hours) or juvenile social interaction, and brains were rapidly removed. The following brain regions were dissected — olfactory bulbs, frontal cortex, striatum, hippocampus, midbrain and brainstem. The dissected tissues were immediately frozen on dry ice and stored at −80°C until analyzed. On the day of assay, the dissected tissue was homogenized in 0.1 mM oxalic acid. The homogenates were centrifuged at 13000×g for 40 min at 4°C. The resulting supernatants were filtrated through a 0.22-µm syringe filter (Millipore, Bedford, MA, USA) then underwent high-performance liquid chromatography (HPLC). The HPLC system consisted of a reverse-phase C18 column (MD-150, RP-C-18, 5 µM, length: 15 cm. ESA, Chelmsford, MA, USA), a high-pressure pump (PM-80, Bioanalytical Systems, West Lafayette, IN) connected with an electrochemical detector (LC-4C) coupled to a reference electrode (Ag/AgCl) and a glassy carbon working electrode, which was set at +750 mV (Bioanalytical Systems). Under an isocratic condition, the solvent of the mobile phase, consisting of 75 mM NaH_2_PO_4_.H_2_O, 1.7 mM 1-Octanesulfonic acid, 25 µM EDTA, 0.72 mM TEA and 10% acetonitrile (pH 3.0; solution degassed for 10 min before use), was pumped and circulated at a flow rate of 1 ml/min in the system. Then 20 µl of sample underwent HPLC.

The concentrations of 5-HT and 5-HIAA in tissue samples were determined with use of an electrochemical detector and the software CSW32 (DataApex, Soubêžnâ, Czech Republic). To quantify the sample peak, each chemical was compared with external standards, which were freshly prepared and injected every 5 sample runs. As an index of 5-HT turnover rate, the 5-HIAA/5-HT ratios were calculated.

### Cross-fostering

Once *Asic3^+/+^* and *Asic3^−/−^* female mice were visibly pregnant, they were housed individually. Pups of *Asic3^+/+^* and *Asic3^−/−^* mice were raised by their biological mother or were cross-fostered to a female of the opposite genotype within 12 h of birth. Cross-fostering, which entailed transferring whole litters, was conducted if both *Asic3^+/+^* and *Asic3^−/−^* dams gave birth within 6 h of one another. An experimenter removed the dams from their home cages and placed them into temporary individual holding cages while the pups were removed from the original nest and placed into the foster dam's nest. The dams were then placed back into their original home cage, which now contained the foster pups. Cross-fostered litters were culled so that each cross-fostered pair had an equal number of pups (10–12, sex ratios ∼1∶1). Litters born more than 6 h apart were raised by their biological mother. Pups remained with their biological or foster mother until they were 21 days old, then were housed in groups of 2 to 5 with same-sex littermates. Social interaction was tested when mice were 5 weeks old.

### Statistical analysis

ABR was analyzed by ANOVA with post-hoc analysis. Pup retrieval, juvenile social behavior and cross-fostering were analyzed by use of ETHOM software[22] and compared by Mann-Whitney U test (non-parametric). All USV data were analyzed by use of Analook software (Titley Elecronics, Ballina, NSW, Australia) and ANOVA with post-hoc analysis. The correlation of pup calls and maternal behaviors was analyzed by regression analyses. Power regression test, logarithmic regression test, linear regression test were used to investigate the correlation between approach latency and call rate, average duration, bout duration respectively. USV emitted during juvenile play was analyzed by chi-square test. Brain neurochemistry between groups was analyzed by unpaired Student's *t* test. A P<0.05 was considered significant.

## Results

### Hearing loss in *Asic3^−/−^* mutant mice

**Figure 1:**
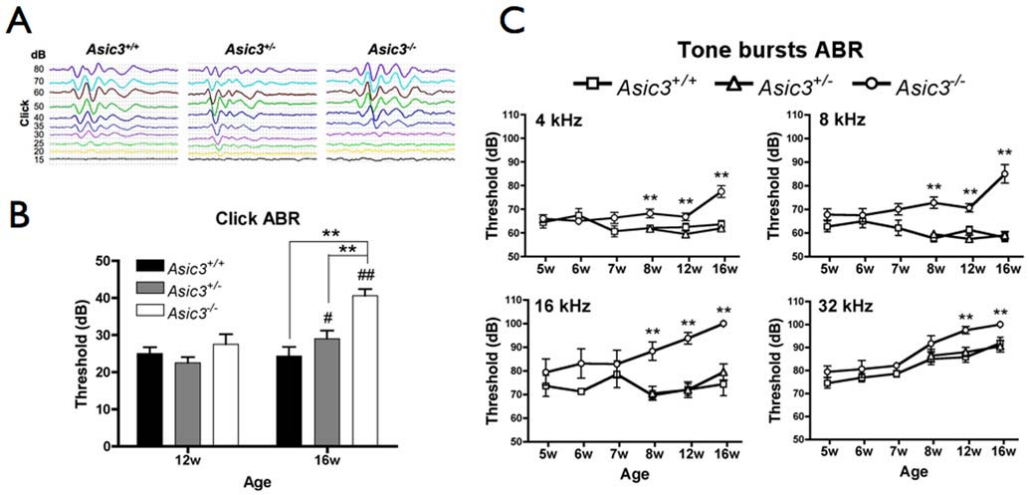
Comparison of hearing thresholds of mice at different ages. (A) Representative click-evoked auditory brainstem responses (ABRs) from mice at 12 weeks of age. (B) Hearing thresholds for click-evoked ABRs between genotypes at 12 and 16 weeks of age. **p<0.01 between *Asic3^−/−^* mice and *Asic3^+/+^* or *Asic3^+/−^* mice of the same age group. #p<0.05, ##p<0.01 between two age groups of the same genotype. (C) *Asic3^−/−^* mice showed significantly higher hearing threshold than *Asic3^+/+^* and *Asic3^+/−^* mice in 4, 8, 16, 32-k frequency of sound at 12∼16 weeks. However there were no differences among genotypes at 5∼7 weeks. Hearing threshold in *Asic3^−/−^* mice significantly increased at 16 weeks than at 8 weeks. *Asic3^+/+^* and *Asic3^+/−^* did not show this age-dependent effect. *p<0.05 and **p<0.01 among genotypes. N = number of 5-week-old mice: *Asic3^+/+^* = 11, *Asic3^−/−^* = 11; 6-week-old mice: *Asic3^+/+^* = 8, *Asic3^−/−^* = 8; 7-week-old mice: *Asic3^+/+^* = 7, *Asic3^−/−^* = 5; 8-week-old mice: *Asic3^+/+^* = 16, *Asic3^+/−^* = 11, *Asic3^−/−^* = 9; 12-week-old mice: *Asic3^+/+^* = 12, *Asic3^+/−^* = 10, *Asic3^−/−^* = 8; 16-week-old mice: *Asic3^+/+^* = 8, *Asic3^+/−^* = 10, *Asic3^−/−^* = 8. Data are mean±SEM.

ABR was used to measure the hearing threshold of mice. Each ABR waveform represented the mean response to 1024 stimulus presentations of clicks and tone bursts of 4, 8, 16 and 32 kHz. A representative click-evoked ABR waveform recorded in mice of different genotypes is shown in Fig. 1*A*. By 12 weeks of age, mice of three genotypes exhibited no significant difference in click-evoked ABR thresholds. *Asic3^+/+^* mice retained normal click-evoked ABR thresholds from 12 to 16 weeks of age. In contrast, *Asic3^−/−^* mice showed an elevated threshold shift at 16 weeks for a significant threshold difference of about 15 dB as compared with *Asic3^+/+^* and *Asic3^+/−^* mice of the same age (Fig. 1*B*).

Hearing thresholds among 4 frequency tone-burst ABR tests were no differences between genotypes at 5∼7 weeks of age (Fig. 1*C*). *Asic3^+/+^* and *Asic3^+/−^* mice showed no statistically significant differences in hearing thresholds among the 4 frequency tone-burst ABR tests at 8, 12 and 16 weeks of age (Fig. 1*C*). In contrast, *Asic3^−/−^* mice at 16 weeks exhibited not only higher tone-burst ABR thresholds at 4 and 8 kHz than mice at 12 weeks but also higher tone-burst ABR thresholds at all 4 frequencies than mice at 8 weeks. In each age group of 12–16 weeks, *Asic3^−/−^* mice showed significant higher thresholds at all 4 frequencies than did the other genotypes (Fig. 1*C*; Fig. S1).

In contrast, *Asic3^−/−^* mice showed normal visual acuity, olfactory acuity, and anxiety levels & locomotion activity based on visual cliff, olfaction habituation tests, and open-field test respectively (Figs. S2, S3 & S4; see Text S1 for the methods).

### Maternal behavior in *Asic3^−/−^* mutant mice

**Figure 2:**
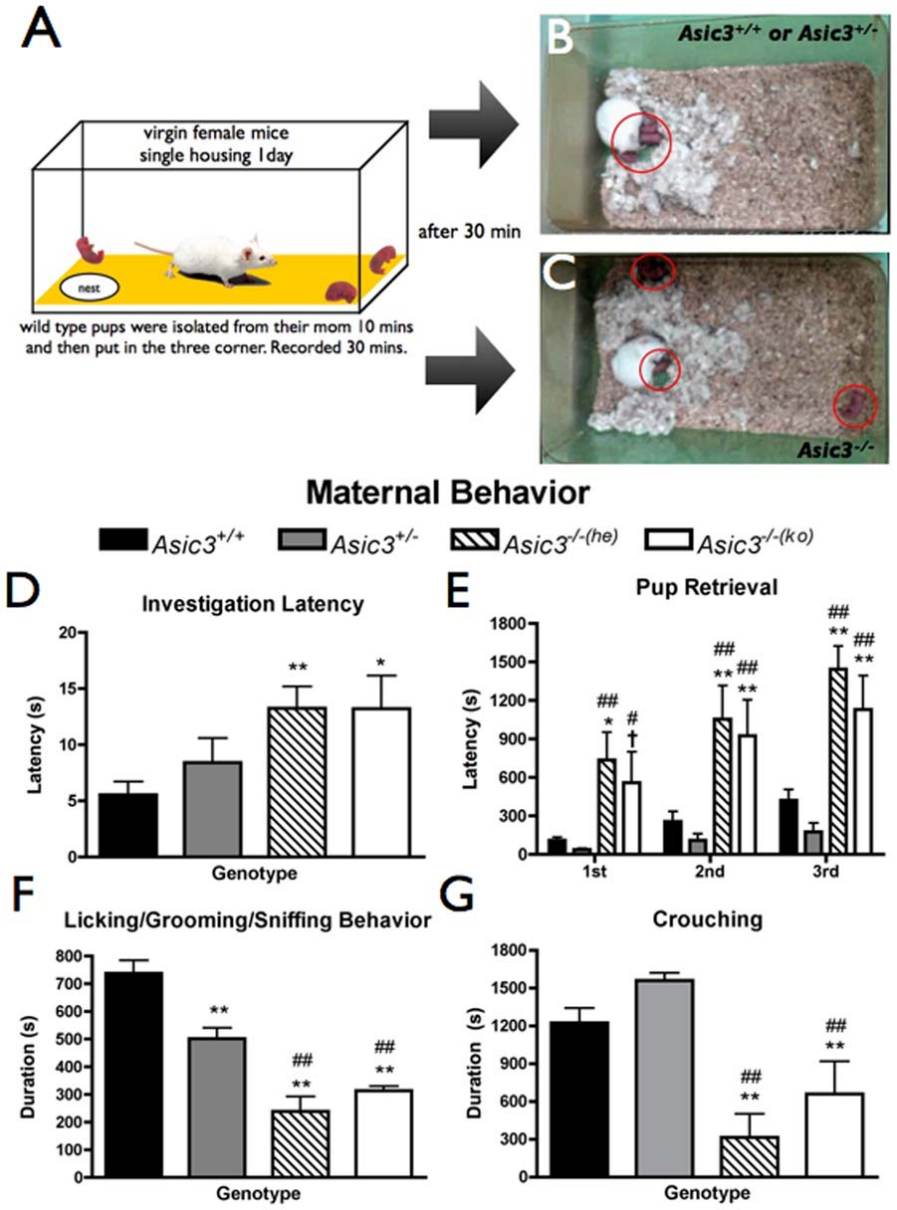
Impaired maternal behaviors of *Asic3^−/−^* mice in pup retrieval assay. (A) Schematic presentation of pup retrieval test. Three pups were put into three corners of the cage away from the nest built by a virgin female mouse. The female mice would investigate the pups immediately after they were transferred into the cage. (B) *Asic3^+/+^* or *Asic3^+/−^* mouse retrieved all pups into the nest and took care of them in 30 min. (C) *Asic3^−/−^* mice retrieved only one pup and left the others out of the nest. (D) Time to investigate pups by female mice. (E) Time to retrieve three pups into the nest. (F) Time spent licking/grooming/sniffing. (G) Time spent crouching. *Asic3^+/+^* mice, n = 9; *Asic3^+/−^* mice, n = 8; *Asic3^−/−(he)^* mice, n = 8; *Asic3^−/−(ko)^* mice, n = 9. *p<0.05, **p<0.01 between *Asic3^+/+^* and other genotypes. #p<0.05, ##p<0.01 between *Asic3^+/−^* and other genotypes. †p = 0.0532 between *Asic3^+/+^* and *Asic3^−/−(ko)^* groups. Data are mean±SEM.

Since hearing baby crying is an important communication between infants and their caregivers, we tested whether the hearing deficit of *Asic3^−/−^* mice could affect their maternal behaviors. Virgin female mice of different genotypes were chosen as caregivers to examine their maternal response to newborn pups (Fig. 2*A*). We used *Asic3^+/+^* pups (1–3 days old) to eliminate the genotype effect of pups. Three pups were isolated from their mothers and put into the caregiver's cage. We tested maternal induction in four groups of mice: *Asic3^+/+^*, *Asic3^+/−^*, *Asic3^−/−(he)^* (*Asic3^−/−^* mice reared by an *Asic3^+/−^* dam) and *Asic3^−/−(ko)^* (*Asic3^−/−^* mice reared by an *Asic3^−/−^* dam) in two consequencial days. *Asic3^−/−^* mice were separated into two groups to verify whether the affected behaviors of maternal induction were due to a genetic or an epigenetic effect. During the 30-min task, *Asic3^+/+^* and *Asic3^+/−^* mice retrieved the three pups to their nests quickly and crouched on them to give warmth (Fig. 2*B*). In contrast, *Asic3^−/−^* mice often retrieved only one pup to their nests, and left the others out of the nest (Fig. 2*C*). Since data on the first day and second day were similar (Fig. S5), the following description was from the results of the second day. *Asic3^−/−(he)^* and *Asic3^−/−(ko)^* mice took significantly longer to investigate the first pup than *Asic3^+/+^* mice (p = 0.0051 and p = 0.0232 respectively; Fig. 2*D*). As well, *Asic3^−/−^* mice (both groups) took more time to retrieve pups than *Asic3^+/+^* and *Asic3^+/−^* mice (Fig. 2*E*). In contrast, *Asic3^+/+^*and *Asic3^+/−^* mice, and *Asic3^−/−(he)^* and *Asic3^−/−(ko)^* mice did not differ in time to investigate and retrieve pups. During the pup retrieval process, *Asic3^−/−^* mice also spent less time in licking/grooming/sniffing the pups than *Asic3^+/+^*and *Asic3^+/−^* mice (*Asic3^+/+^* vs. *Asic3^−/−(he)^* p = 0.0005; *Asic3^+/+^* vs. *Asic3^−/−(ko)^* p = 0.0003; *Asic3^+/−^* vs. *Asic3^−/−(he)^* p = 0.0046; *Asic3^+/−^* vs. *Asic3^−/−(ko)^* p = 0.0008; Fig. 2*F*). Notably, *Asic3^+/−^* mice also spent less time than *Asic3^+/+^* mice licking/grooming/sniffing the pups (p = 0.0003).

Although all *Asic3^+/+^*and *Asic3^+/−^* mice retrieved three pups in 15 min, only 58.8% *Asic3^−/−^* mice retrieved three pups, and most took more than 20 min to retrieve all pups. For mice that completed the pup retrieval, *Asic3^−/−^* mice (both groups) showed less crouching behaviors than other genotypes (Fig. 2*G*). Besides showing less capability of pup retrieval, *Asic3^−/−^* mice (45% of *Asic3^−/−(ko)^* and 12.5% of *Asic3^−/−(he)^*) also caused pup injury or even death when they investigated or retrieved pups (Fig. S6); no *Asic3^+/+^* and *Asic3^+/−^* mice caused pup injury. Thus, *Asic3^−/−^* virgin mice showed defects in maternal induction, whether reared by *Asic3^+/−^* or *Asic3^−/−^* dams, which infers that a genetic rather than an epigenetic effect dominates the maternal deficit in *Asic3^−/−^* mice. The poor pup retrieval in *Asic3^−/−^* mice was associated with their age-dependent hearing impairment. Comparing with the younger *Asic3^−/−^* mice (8–12 weeks), the older ones (12–16 weeks) took longer time to retrieve all pups on day 1 and spent less time for licking/grooming/sniffing pups on day 2 (Fig. S7).

In consistent with the results of *Asic3^−/−^* virgin female mice, we also found that *Asic3^−/−^* dams performed poor pup retrieval for their own pups (Fig. S8 & Movie S1; see Text S1 for the method). As well, *Asic3^−/−^* dams showed high incidence of infanticide. *Asic3^−/−^* dams had significantly less weaned pups than *Asic3^+/−^* or *Asic3^+/+^* dams (Fig. S9). Nevertheless, *Asic3^−/−^* dams were capable of taking care of their own pups, since their offspring had a normal body weight at the weaned ages as compared with those raised by *Asic3^+/−^* or *Asic3^+/+^* dams (Fig. S10).

### Maternal response to pup calls in *Asic3^−/−^* mice

**Figure 3:**
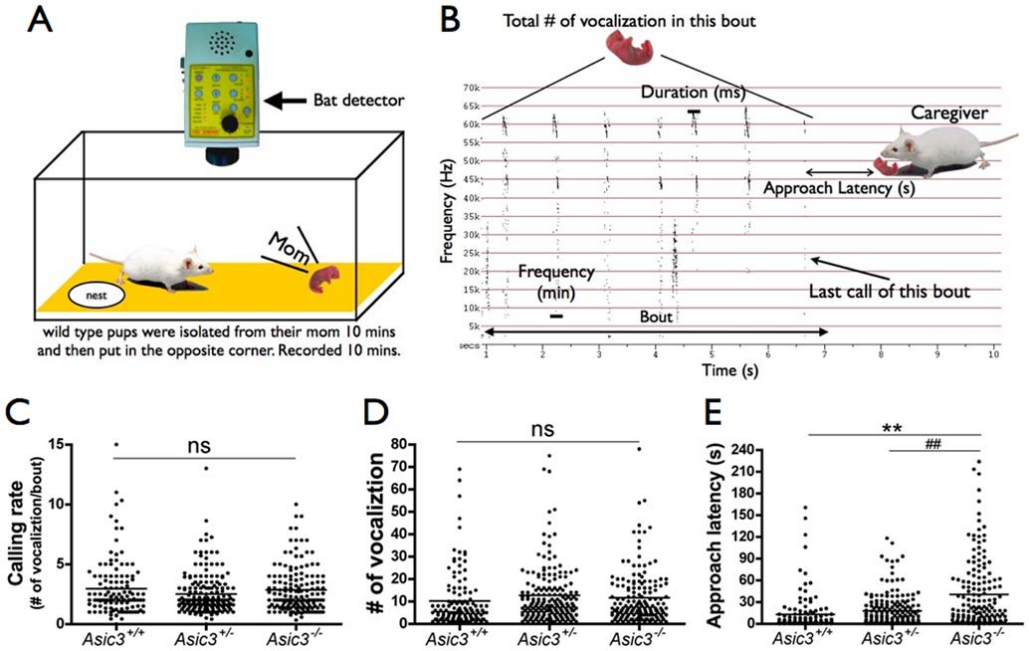
Approach latency of virgin mice for an isolated pup's USV. (A) Schematic presentation of the method used to record pup USV and female maternal behavior. (B) A representative sonogram of a bout emitted from an isolated pup. The parameters used in pup USV and pup retrieval are defined in the sonogram. (C) The call rate emitted by wild-type pups that confronted caregivers of three *Asic3* genotypes. (D) The total number of vocalizations emmited by wild type pups that confronted caregivers of three *Asic3* genotypes. (E) The approach latency after a bout. Bout number in *Asic3^+/+^* group was 124; *Asic3^+/−^* group was 179; *Asic3^−/−^* group was 163. **p<0.01 between *Asic3^−/−^* and *Asic3^+/+^* groups. ##p<0.01 between *Asic3^−/−^* and *Asic3^+/−^* groups. ns, not significant.

We next asked whether the poor pup retrieval of *Asic3^−/−^* mice was due to a hearing deficit in response to pup USV. We simultaneously recorded pup USV and caregivers' pup retrieval behaviors (Fig. 3*A*). In the USV recording, pups emitted discrete USVs in a bout, ranging from 1 to 15 s (Fig. 3*B*). We identified 124, 179, 163 discrete bouts of pup calls in *Asic3^+/+^*, *Asic3^+/−^*, *Asic3^−/−^* caregiver groups, respectively (n = 10 for each group of mice). Pups emitted USVs with similar quality and quantity, in terms of the calling rate and total number of vocalizaitons, for different genotypes of caregivers (Fig. 3*C, D*). However the pups emitted longer bout durations for *Asic3^+/−^* and *Asic3^−/−^* virgin mice than for *Asic3^+/+^* mice (Fig. S11). *Asic3^+/+^* and *Asic3^+/−^* mice showed quicker response (shorter approach latency) to pup calls than *Asic3^−/−^* mice (12.91±2.38 and 17.69±2.05 s vs. 40.28±4.45 s) (Fig. 3*E* & Movie S2).

**Figure 4:**
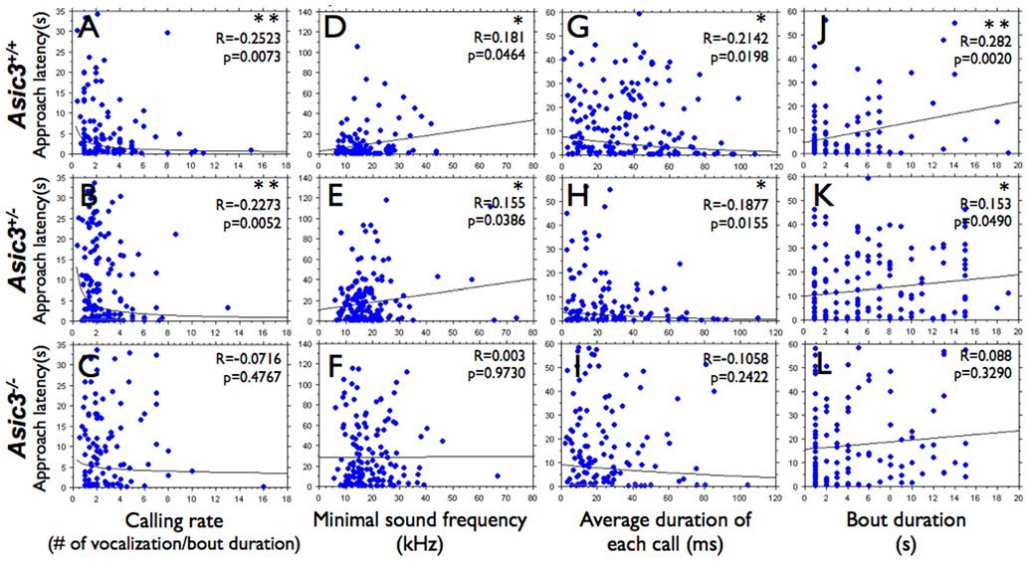
Regression analysis of approach latency with parameters of pup vocalization in caregivers of three *Asic3* genotypes. (A, B, C) Correlation of approach latency with calling rate in *Asic3^+/+^* mice (bout n = 112, R = −0.2523, p<0.01), *Asic3^+/−^* mice (bout n = 150, R = −0.2273, p<0.01) and *Asic3^−/−^* mice (bout n = 101, R = −0.0.0716, p = 0.4767) with cut-off time of approach latency of 35 s (power regression test). (D, E, F) Correlation of approach latency with average minmal sound frequency in a bout in *Asic3^+/+^* mice (R = 0.181, p<0.05, n = 121), *Asic3^+/−^* mice (R = 0.155, p<0.05, n = 179) and *Asic3^−/−^* mice (R = 0.003, p = 0.9730, n = 149) with cut-off time of approach latency of 120 s (linear regression test). (G, H, I) Correlation of approach latency with average duration of each call in a bout in *Asic3^+/+^* mice (R = −0.2142, p<0.05, n = 118), *Asic3^+/−^* mice (R = −0.1877, p<0.05, n = 166) and *Asic3^−/−^* mice (R = −0.1058, p = 0.2422, n = 124) with cut-off time of approach latency of 60 s (exponential regression test). (J, K, L) Correlation of approach latency with bout duration in *Asic3^+/+^* mice (R = 0.282, p<0.01, n = 118), *Asic3^+/−^* mice (R = 0.153, p<0.05, n = 166) and *Asic3^−/−^* mice (R = 0.088, p = 0.3290, n = 124) with cut-off time of approach latency of 60 s (linear regression test). *p<0.05, **p<0.01.

We next conducted regression analyses to determine the correlation of pup calls with approach latency. We used power regression analysis to investigate the correlation between pup calling rate and approach latency. At a cut-off time of 35 s for approach latency, pup calling rate was negatively correlated with approach latency in *Asic3^+/+^* (R = −0.2523; p = 0.0073; n = 112; Fig. 4*A*) and *Asic3^+/−^* (R = −0.2273; p = 0.0052; n = 150; Fig. 4B) but not *Asic3^−/−^* mice (R = −0.0716; p = 0.4767; n = 101; Fig. 4C). Pup calling rate was also negatively correlated with approach latency for *Asic3^+/+^* and *Asic3^+/−^* mice at cut-off approach times of 18.5∼37, 45 or 118∼145.7 s (data not shown). Thus, *Asic3^+/+^* and *Asic3^+/−^* mice responded to pups' rush calls but *Asic3^−/−^* mice did not. Next, we used linear regression test to investigate the correlation between average minimal sound frequency (Fmin) of each vocalization in a bout and approach latency. At a cut-off time of 120 s for approach latency, Fmin showed a significant positive correlation with approach latency for *Asic3^+/+^* (R = 0.181; p = 0.0464; n = 121; Fig. 4D) and *Asic3^+/−^* mice (R = 0.155; p = 0.0386; n = 179; Fig. 4E) but no significant correlation for *Asic3^−/−^* mice (R = 0.003; p = 0.9730; n = 149; Fig. 4F). Thus, the lower the Fmin (with frequency spectrum expanded to the ranges of wriggling calls), the sooner the *Asic3^+/+^* and *Asic3^+/−^* mice responded at cut-off approach times of 45∼118 s. Next, we used exponential regression test to investigate the correlation between average duration of each USVs in a bout and approach latency. At a cut-off time of 60 s for approach latency, average duration showed a significant negative correlation with approach latency for *Asic3^+/+^* (R = −0.2142; p = 0.0198; n = 118; Fig. 4G) and *Asic3^+/−^* mice (R = −0.1877; p = 0.0155; n = 166; Fig. 4H) but no significant correlation for *Asic3^−/−^* mice (R = −0.1058; p = 0.2422; n = 124; Fig. 4I). Thus, the longer the USV duration, the sooner the *Asic3^+/+^* and *Asic3^+/−^* mice responded at cut-off approach times of 34.2∼145.7 s. Moreover, bout duration was positively correlated with approach time in *Asic3^+/+^* and *Asic3^+/−^* mice at cut-off approach times of 33.4∼36.9 or 47.9∼59.4 s (linear regression test). At the cut-off time of 60 s, bout duration and approach latency showed a significant positive correlation for *Asic3^+/+^* (R = 0.282; p = 0.0020; n = 118; Fig. 4J) and *Asic3^+/−^* mice (R = 0.153; p = 0.0490; n = 166; Fig. 4K) but no significant correlation for *Asic3^−/−^* mice (R = 0.088; p = 0.3290; n = 124; Fig. 4*L*). In contrast, the number of vocalizations, and maximum and mean sound frequency (Fmax and Fmean) of USVs in a bout showed no correlation with approach latency in the three genotypes (data not shown). Thus, *Asic3^+/+^* and *Asic3^+/−^* caregivers rushed to pup's calls when they received a high calling rate, a low Fmin, a long USV duration, or a short bout from pups but *Asic3^−/−^* mice had no significant response to pups' calls in any circumstance. These results indicate that ASIC3 is involved in mothers receiving sensation of USV calls from pups; mice lacking *Asic3* cannot provide a prompt response to a pup's need.

### Offspring social development

**Figure 5:**
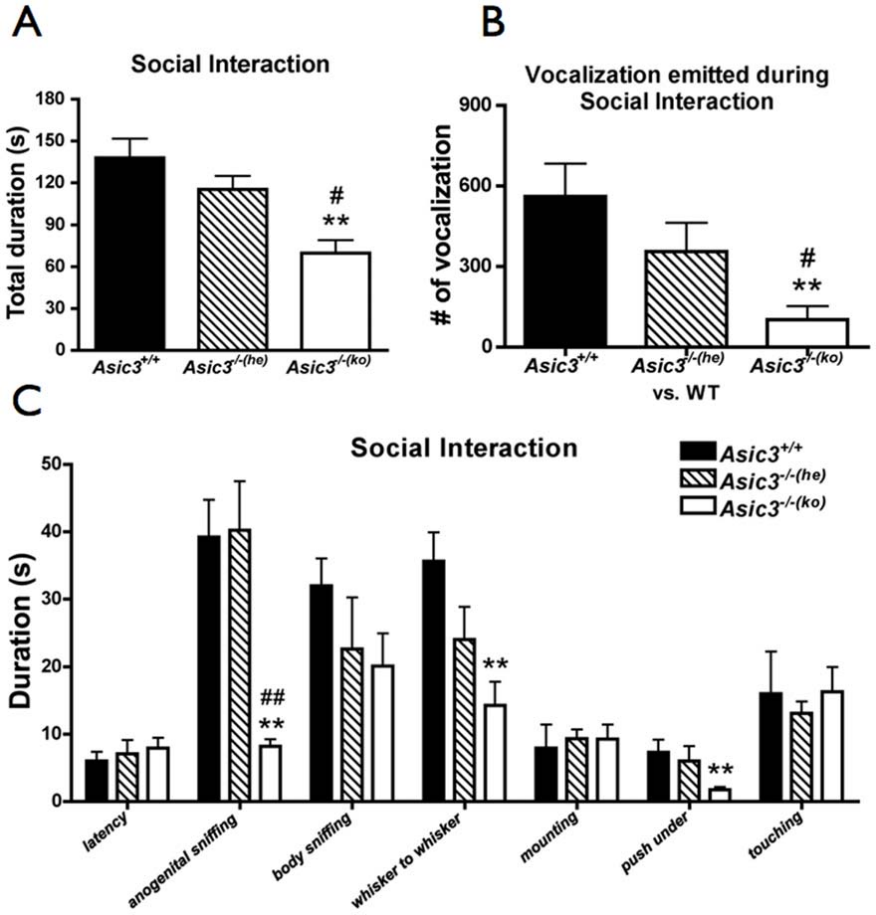
Comparison of social activity in juvenile social behaviors among *Asic3^+/+^*, *Asic3^−/−(he)^*, and *Asic3^−/−(ko)^* mice. (A) Time in social interaction with a wild-type (WT) mouse for juvenile *Asic3^−/−(ko)^* mice (n = 10; from homozygous breeding), *Asic3^+/+^* (n = 10) and *Asic3^−/−(he)^* mice (n = 8). (B) Number of vocalizations emitted during social interaction for *Asic3^−/−(ko)^*-WT pair and other pairs (n = 7 for each group). (C) Affiliative behaviors in juvenile social activities of *Asic3^+/+^*, *Asic3^−/−(he)^* and *Asic3^−/−(ko)^* mice. **p<0.01 between *Asic3^+/+^* and other genotypes. #p<0.05, ##p<0.01 between *Asic3^−/−(he)^* and other genotypes. Data are mean±SEM.

*Asic3^−/−^* mice were separated into two groups depending on their being reared by an *Asic3^+/−^* or *Asic3^−/−^* dam to test juvenile social interaction in mice. *Asic3^−/−(ko)^* mice spent less time in social interaction with an unfamiliar control mouse than *Asic3^+/+^* (p = 0.0015) and *Asic3^−/−(he)^* mice (p = 0.0209) (Fig. 5*A*). The duration of behaviors of anogenital sniffing, whisker to whisker, and push under was less in *Asic3^−/−(ko)^* mice than in *Asic3^+/+^* mice (p = 0.0003, 0.0025, 0.0019 respectively; Fig. 5*C*). As well, *Asic3^−/−(he)^* and *Asic3^−/−(ko)^* mice showed a significant difference in duration of anogenital sniffing (p = 0.0004; Fig. 5*C*), but *Asic3^−/−(he)^* and *Asic3^+/+^* mice showed no difference in affiliative behaviors of anogenital sniffing (p = 0.9292), body sniffing (p = 0.0914), whisker to whisker (p = 0.0914), mounting (p = 0.1551), push under (p = 0.3743) or touching (p = 0.4229). Thus, the inadequate maternal care of the *Asic3^−/−^* dams hindered the social development of their offspring.

### Vocalization during juvenile social interaction

**Figure 6:**
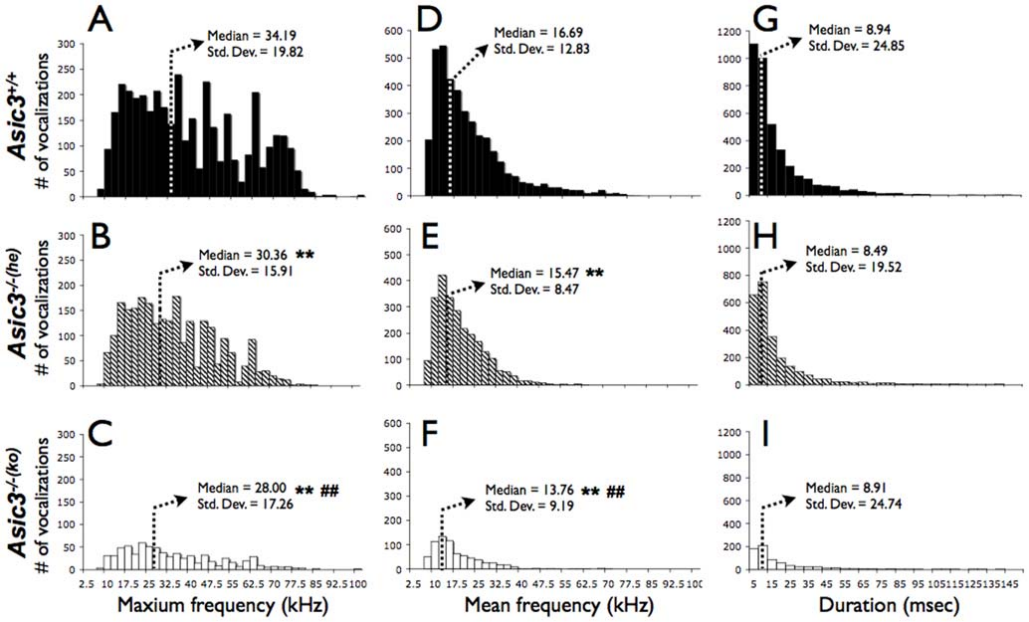
Sonographic characteristics of USVs during social interaction of juvenile genotype mice with wild-type (WT) mice. (A, B, C) Histograms of maximum frequency (Fmax) of USV emission patterns for *Asic3^−/−(he)^*–WT, *Asic3^−/−(ko)^*–WT, and *Asic3^+/+^*-WT pairs. (D, E, F) Histograms of mean frequency (Fmean) of USV emission patterns for *Asic3^−/−(he)^*–WT and *Asic3^−/−(ko)^*–WT, and *Asic3^+/+^*-WT pairs. (G, H, I) USV duration among three groups. **p<0.01 with *Asic3^+/+^*-WT pair. ##p<0.01 between *Asic3^−/−(he)^*–WT and *Asic3^−/−(ko)^*–WT pairs.

Vocalization recording during social interaction can be an index of mice communication. *Asic3^−/−(ko)^* mice emitted less vocalization than *Asic3^−/−(he)^* mice and *Asic3^+/+^* mice on encountering an unfamiliar mouse during 15 min of juvenile play (p = 0.0253 and 0.0073 respectively; Fig. 5*B*). The null mutation of *Asic3* did not suppress vocalization in juvenile social interaction, because *Asic3^−/−(he)^* mice and *Asic3^+/+^* mice did not differ in total number of vocalizations (Fig. 5*B*). To further examine whether the genetic effect of *Asic3* knockout affected the vocalization profile in mice, we analyzed the sonographic characteristics of vocalization during the social interaction of juvenile mice (Fig. 6). Fmax (Fig. 6*A, B, C*) and Fmean (Fig. 6*D, E, F*) of vocalizations produced by *Asic3^+/+^* mice showed different distribution than those emitted by *Asic3^−/−(he)^* mice (Fmax: χ^2^ = 260.739, d.f. = 1, p<0.0001; Fmean: χ^2^ = 166.433, d.f. = 1, p<0.0001) and *Asic3^−/−(ko)^* mice (Fmax: χ^2^ = 99.131, d.f. = 1, p<0.0001; Fmean: χ^2^ = 81.189, d.f. = 1, p<0.0001). *Asic3^−/−(ko)^* mice also differed from *Asic3^−/−(he)^* mice in terms of Fmax (χ^2^ = 57.830, d.f. = 1, p = 0.0034; Fig. 6*B, C*) and Fmean (χ^2^ = 53.859, d.f. = 1, p = 0.0004; Fig. 6*E, F*). In contrast, vocalization duration was similar among all mice groups (Fig. 6G, *H, I*). The three groups of mice showed distinguishable patterns of ultrasonic communication in sonograms. Thus, the ultrasonic communication of juvenile mice was influenced by ASIC3 and affected by both genetic and epigenetic modulation.

### Neuronal activity during juvenile social interaction

**Figure 7:**
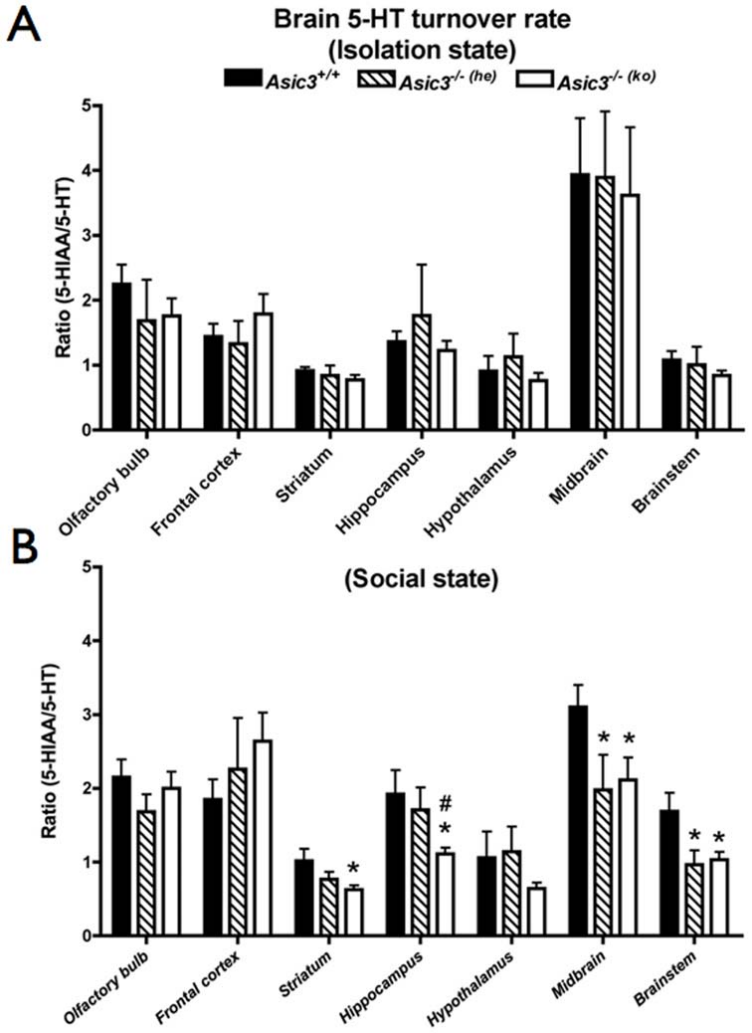
Brain neurochemical changes (5-HT turnover) in brain regions after juvenile social interaction. (A) 5-HT turnover rates in isolation state. (B) 5-HT turnover rates after social interaction. The number of experiments (n) is indicated above each bar. *p<0.05, between *Asic3^+/+^* and other genotypes. #p<0.05, between *Asic3^−/−(he)^* and *Asic3^−/−(ko)^* mice. Data are mean±SEM.

The serotonergic system is closely related to social behaviors in mammals. To further understand whether the socially deficient phenotypes of *Asic3^−/−(ko)^* mice were due to alteration of the central serotonergic transmission, we examined the 5-HT turnover rates in different brain regions from mice in isolation state and after juvenile social interaction. In isolation state, 5-HT turnover rates in all brain regions were not different between groups (Fig. 7A). After social interaction, the 5-HT turnover rate was lower in striatum (p = 0.0233), hippocampus (p = 0.0255), midbrain (p = 0.0361), and brainstem (p = 0.0181) of *Asic3^−/−(ko)^* than *Asic3^+/+^*mice (Fig. 7B). In contrast, the 5-HT turnover rate of *Asic3^−/−(he)^* mice was only lower than *Asic3^+/+^* in midbrain (p = 0.0392) and brainstem (p = 0.0233), indicating the genetic effect on these two brain regions. In comparing two *Asic3^−/−^* groups, the hippocampal 5-HT turnover rate was lower in *Asic3^−/−(ko)^* than in *Asic3^−/−(he)^* mice (p =  0.0329). Thus, 5-HT release showed an epigenetic effect in mice. The three groups did not show differences in 5-HT turnover in other brain regions. On encountering unfamiliar mice, the brains of *Asic3^−/−(ko)^* mice released less 5-HT than those of *Asic3^+/+^* and *Asic3^−/−(he)^* mice.

### Cross-fostering study

**Figure 8:**
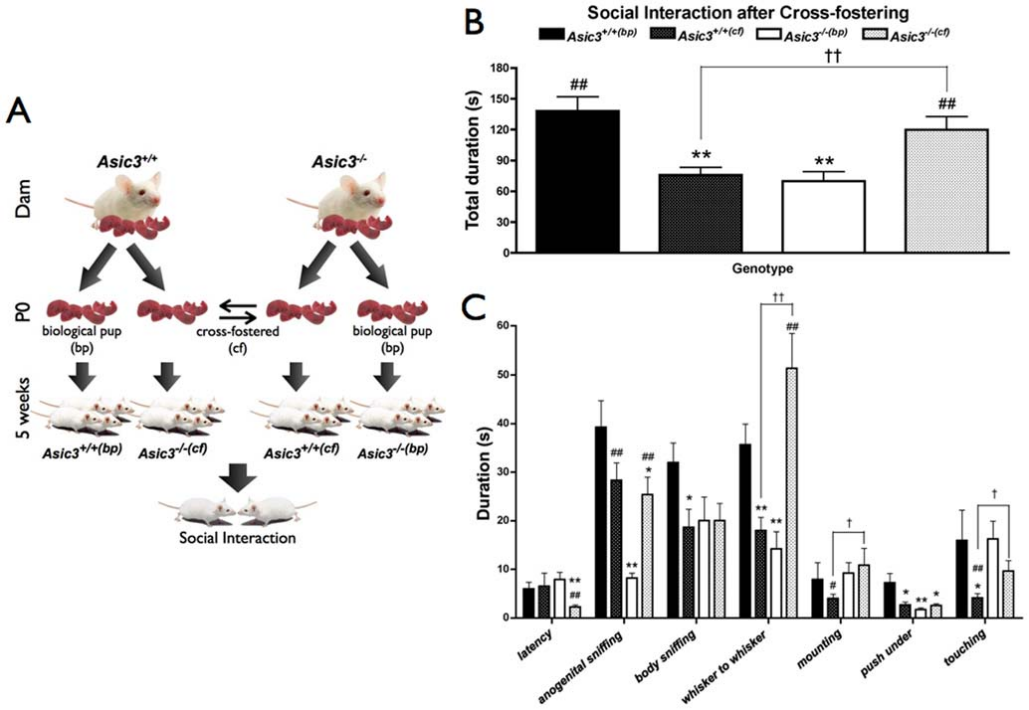
Effect of cross-fostering on social interaction of juvenile mice. (A) Experimental scheme used to raise mice in a cross-fostering paradigm. The biological (not cross-fostered) pups (bp) have the same genotype as the dam raising them, and the cross-fostered pups (cf) have a genotype different from that of the dam raising them. (B) Social interaction activity in juvenile among four groups of mice. (C) Affiliative behaviors in juvenile among four groups of mice. *Asic3^+/+(bp)^* n = 10; *Asic3^+/+(cf)^* n = 10; *Asic3^−/−(bp)^* n = 10; *Asic3^−/−(cf)^* n = 9. *p<0.05, **p<0.01 between *Asic3^+/+(bp)^* mice and other groups. #p<0.05, ##p<0.01 between *Asic3^−/−(bp)^* mice and others. ††p<0.01 between *Asic3^+/+(cf)^* and *Asic3^−/−(cf)^* mice. Samples sizes represent mice from 3 litters. Data are mean±SEM.

We further examined the maternal effect on offspring social development by cross-fostering *Asic3^+/+^* pups to *Asic3^−/−^* dams and *Asic3^−/−^* pups to *Asic3^+/+^* dams at the day of birth. Accordingly, we defined four groups of mice for examination of juvenile social interaction. Two groups of mice were biological pups — *Asic3^+/+(bp)^* (biological pups of *Asic3^+/+^* mice) and *Asic3^−/−(bp)^* (biological pups of *Asic3^−/−^* mice) raised by the same genotype dam; the other two groups were cross-fostered pups — *Asic3^+/+(cf)^* (cross-fostered pups of *Asic3^+/+^* mice) and *Asic3^−/−(cf)^* (cross-fostered pups of *Asic3^−/−^* mice) raised by opposite-genotype mice (Fig. 8*A*). Cross-fostering had a pronounced effect on the social development of mice. *Asic3^+/+(cf)^* mice showed reduced total activity of juvenile social behaviors as compared with *Asic3^+/+(bp)^* mice (p = 0.0015; Fig. 8*B*). Cross-fostering reduced behaviors of body sniffing, whisker to whisker, push under, and touching in *Asic3^+/+(cf)^* mice (p = 0.0413; 0.0052; 0.0126; 0.0284, respectively; Fig. 8*C*). However, *Asic3^+/+(cf)^* mice showed no difference from *Asic3^−/−(bp)^* mice in total social activity, but these two mice had very different juvenile social behaviors. *Asic3^+/+(cf)^* mice had higher activities of anogenital sniffing (p = 0.0002) but lower activities of mounting (p = 0.0102) and touching (p = 0.0004) than *Asic3^−/−(bp)^* mice (Fig. 8*C*). In contrast, cross-fostering to the wild-type dam rescued *Asic3^−/−(cf)^* mice from the socially deficient phenotype. *Asic3^−/−(cf)^* mice spent significantly more time on juvenile social behaviors than did *Asic3^−/−(bp)^* and *Asic3^+/+(cf)^* mice (p = 0.0071 and p = 0.0090 respectively; Fig. 8*B*). Especially, cross-fostering largely improved behaviors of anogenital sniffing and whisker to whisker in *Asic3^−/−(cf)^* mice as compared with *Asic3^−/−(bp)^* mice (p = 0.0002 and 0.0008 respectively). Also, *Asic3^−/−(cf)^* mice showed shorter latency in interacting with the control partner than did *Asic3^+/+(bp)^* and *Asic3^−/−(bp)^* mice (p = 0.009 and 0.002 respectively). Therefore, the social development of *Asic3^+/+^* pups was hindered when they were cross-fostered to *Asic3^−/−^* dams. However, the social development of *Asic3^−/−^* pups could be improved when they were cross-fostered to *Asic3^+/+^* dams. The maternal deficit of *Asic3^−/−^* dams but not *Asic3* genotype impaired the social development of young pups. Overall, the cross-fostering study revealed that maternal care notably affects offspring social development.

## Discussion

Our study revealed that loss of ASIC3-dependent auditory sensation in female mice has a profoundly negative effect on their maternal behaviors and thus impairs the social development of their pups. Female mice lacking *Asic3* showed impaired low- and high-frequency hearing, which resulted in their lack of response to pup calls and impaired maternal behaviors of retrieving pups to nests and providing care. Mouse pups receiving less care developed socially deficient phenotypes as shown in their juvenile social behaviors, and reduced vocalization and serotonergic nerve activity during social interaction. However, *Asic3^−/−^* pups could achieve normal social development when the caregivers were *Asic3^+/+^* or *Asic3^+/−^* female mice.

ASIC2 and ASIC3 are functionally expressed in cochlear spiral ganglia[17], [23]. However, knockout mouse-model studies showed that neither ASIC2 nor ASIC3 is required for normal hearing sensitivity[16], [17], [24]. ASIC2 contributes to suprathreshold hearing function by specifically affecting a noise-induced temporary threshold shift[16]. Loss of ASIC3, in contrast, results in an age-dependent hearing loss[17]. Our click-evoked ABR data were consistent with those of a previous study showing that *Asic3^−/−^* mice at 12 weeks retained a normal click-evoked ABR threshold. However, on analyzing specific tone-burst ABRs, we found that ASIC3 may be involved in the receipt of tone stimuli, especially at high frequencies. In the current study, *Asic3^−/−^* mice showed elevated hearing threshold at 4, 8, 16 and 32 kHz as early as 8 weeks of age, when the click-evoked ABR threshold remained stable. Thus, hearing impairment at high frequencies may occur as early as 8 weeks in mice lacking *Asic3*. Moreover, the auditory deficits in *Asic3^−/−^* mice gradually worsened with age and progressed to near deafness at high and ultrasonic frequencies (16, 32 kHz) by 16 weeks of age. Taken together, our data demonstrate an ASIC3-dependent hearing-loss pattern in mice that is related to age and high frequency and may be linked to a deficit on receiving USV.

Communication in mice, especially the pup-mother interaction,[11], [13] largely depends on wriggling calls (2–20 kHz) and USV (above 20 kHz)[25], [26]. Loss of high-frequency sound hearing in *Asic3^−/−^* foster mice implies that they may have problems communicating with pups and fail to fulfill the pups' needs. The pup-mother interaction depends on two important components — cues emitted from pups and sensation in the mother[27], [28]. Hearing infant crying followed by an appropriate response is a basic communication of the infant-mother interaction, especially in rodents[11], [13], [29], [30]. A pup in stress emits USV; the dam receives a sensation of the pup's USV and then approaches the pup to determine what is needed. While this theory is well accepted, the mothers' maternal behaviors may also tune the pup's USV calls[13], [30], [31]. We used virgin female mice to demonstrate the effect of ASIC3-dependent hearing deficit on maternal induction when the virgin mice first contact the newborn pups. This method could eliminate the effect of cortical entrainment to pup communication calls in mothers[32]. *Asic3^−/−^* virgin mice showed deficits in pup retrieval and pup care as compared with *Asic3^+/+^*and *Asic3^+/−^* virgin mice. This maternal deficit in *Asic3^−/−^* female mice was strongly linked to their hearing deficit because their approach behaviors were not significantly correlated with pup USV as were *Asic3^+/+^* and *Asic3^+/−^* mice behaviors. Moreover, *Asic3^−/−^* mother mice often injured their own pups, a pattern not found in *Asic3^+/+^*and *Asic3^+/−^* mice. Perhaps *Asic3^−/−^* mice could not properly hear the distress calls emitting from pups during the care process. By analogy, this phenomenon is also observed in pups raised by deaf rats but not blind rats[28]. Interestingly, the rate of *Asic3^−/−(he)^* mothers injuring their own pups was less than that of *Asic3^−/−(ko)^* mothers. Thus, postnatal care could improve the maternal behaviors of mice with hearing deficit. According to attachment theory, parents' states of mind are transmitted to the child[3], [6], which may explain why *Asic3^−/−(he)^* mice, raised by Asic3^+/+^ mice, were more careful in taking care of pups and thus prevented hurting them than *Asic3^−/−(ko)^* mice.

Social development is controlled by not only genes but also by the external environment[33]–[35]. Adult behaviors can be modified by early experiences during infancy[36]. Especially, maternal care provides an epigenetic effect to the social development of offspring[37]. Rodent offspring that received low levels of licking or grooming from their mothers showed decreased 5-HT levels in the midbrain and hippocampus[38], [39]. A high level of 5-HT in the hippocampus was shown to increase nerve growth factor1-A level and induce glucocorticoid receptor gene expression during development, which alters animals' adaptation to stress in adulthood[40]. In addition, early social enrichment (offspring benefiting from the sharing of maternal care-giving by multiple females in a single nest) increases the brain's neurotrophin levels in young animals and shapes social competencies in adulthood[7]. Our results are consistent with these previous results showing that the maternal hearing deficits of *Asic3^−/−^* mice result in impaired social development in their pups. In contrast, *Asic3^−/−^* pups could develop normal social ability if raised by *Asic3^+/−^* mice or cross-fostered to *Asic3^+/+^* mothers. With the same genotype but being cared for by a mother with an *Asic3^−/−^* or *Asic3^+/−^* genotype, juvenile *Asic3^−/−(ko)^* male mice displayed lower social interaction, especially in anogenital sniffing, and emitted less vocalization than *Asic3^−/−(he)^* male mice in juvenile social behaviors.

USV emitted during social interaction can be a useful index to indicate communication between two mice[26], [30], [41]. Both *Asic3* knockout groups emitted USV patterns different from that of *Asic3^+/+^* mice, but the USV pattern of *Asic3^−/−(he)^* mice was closer to that of *Asic3^+/+^* mice and had higher maximum and mean frequencies than *Asic3^−/−(ko)^* mice. Enhanced maternal care of *Asic3^−/−^* mice could increase the number and the mean frequency of vocalizations, which further reveals the value of maternal care. In addition, the epigenetic effect of maternal care was found in brain neurochemical features, in 5-HT turnover rate, for instance. After 15 min of social interaction, juvenile *Asic3^−/−(he)^* mice showed levels of 5-HT turnover rate in the hippocampus similar to that of *Asic3^+/+^* mice but significantly higher turnover rate than that of *Asic3^−/−(ko)^* mice. The alteration in brain 5-HT transmission affects the social behaviors of mice[42], [43]. Therefore the low social interaction and USV emission of *Asic3^−/−(ko)^* mice could result from the alteration of serotonin transmission in the hippocampus.

We further tested the ASIC3-dependent effect in maternal care on social development by cross-fostering the offspring of *Asic3^+/+^* and *Asic3^−/−^* dam at the day of birth. This method has been extensively adopted for investigating epigenetic effects on offspring[44]–[48]. However, most cross-fostering research has focused on anxiety, anxiety-related neurochemical changes, and stress response. None has tested the cross-fostering effect on social interaction. In cross-fostering the offspring of *Asic3^+/+^* and *Asic3^−/−^* dams, the total activity of juvenile social behaviors was reversed. *Asic3^+/+^* offspring showed decreased social interaction when they were cross-fostered to *Asic3^−/−^* dams, whereas *Asic3^−/−^* offspring showed elevated social interaction when cross-fostered to *Asic3^+/+^* dams. However, the specific social behaviors of cross-fostered offspring were not exactly identical to those of the opposite genotype. Anogenital sniffing behaviors were increased, but mounting and touching behaviors were decreased in cross-fostered *Asic3^+/+^* offspring as compared with biological *Asic3^−/−^* offspring. The social latency, push under and anogenital sniffing behaviors were lower in cross-fostered *Asic3^−/−^* offspring than in biological *Asic3^+/+^* offspring. The most dramatic change of social behaviors altered in cross-fostered offspring was the whisker-to-whisker behavior. Cross-fostering largely increased the frequency and duration of the behavior whisker to whisker in *Asic3^−/−^* offspring. Though cross-fostering itself could result in stress for both dams and pups, the effect is subtle in mice[46], [49] and should be minimal in this study. Even if the stress existed, we still observed that the *Asic3^−/−(cf)^* pups benefited from the cross-fostering by an *Asic3^+/+^* dam (Fig. 8B). Complex sensory and motor modalities and complicated central processing are involved in social behaviors in animals[50]. Here we demonstrated that ASIC3-dependent sensory deficit could affect social development and modulate the complexity of social behaviors through epigenetic maternal caring.

One may still argue that the hearing cannot be totally responsible for the maternal and social hehavior deficits found in this study. Indeed, *Asic3^+/−^* mice performed normal hearing acuity, so their licking/grooming/sniffing phenotype might be also contributed by other ASIC3-dependent sensory function (Figs. 1 & 2F). Previous studies have shown that null mutation of *Asic3* resulted in abnormal mechanosensation and nociception in mice[21], [51], [52], which may then affect the licking/grooming/sniffing behaviors. Though *Asic3^+/−^* females showed maternal deficits (licking/grooming/sniffing behaviors) that were hearing-independent, the *Asic3^−/−(he)^* pups raised by them had normal social development (Fig. 5). Therefore, these data further support that the hearing deficit of *Asic3^−/−^* mice was responsible, though not exclusively, for the maternal deficit and pup social development.

In conclusion, ASIC3-dependent hearing by mouse mothers of pups' calls plays an important role in maternal behaviors in mice. Mice lacking *Asic3* cannot receive distress calls from pups and thus fail to provide proper care. Pups who received less care showed less sociability with peers when they grew up. Hearing a baby's cries and responding to the cries helps form a secure attachment between infant and mother[4], [53]. Secure attachment is essential for early social development in humans. However, how this secure attachment affects social development later on, in adolescence or even adulthood, is still not clear. In this study, we used a hearing-deficit mouse model to demonstrate a pronounced effect of maternal care on adolescent social development. Our research has implications for assisting parents with hearing deficit help their children achieve normal social development. Also, our results provide a new therapeutic aspect for psychological diseases related to the peripheral sensory pathway.

## Supporting Information

Figure S1 Representative 4-, 8-, 16- and 32-kHz tone burst-evoked auditory brainstem responses (ABRs) from mice at 12 weeks of age. Asic3−/− mice showed elevated hearing threshold with 4 and 8 kHz as compared with other genotypes. With 16- and 32-kHz tone bursts, Asic3−/− mice were nearly deaf, whereas other genotypes showed normal hearing.
(0.40 MB TIF)

Figure S2 Visual cliff. (A) Apparatus: left side is the “safe” zone; right side is the “cliff” zone. Mice with normal vision will step down to the safe zone instead of cliff zone. Each mouse had 10 trials to choose the zone. The box were turned 180° after 5 trials to eliminate the memory effect. (B) 85.53% of ASIC3+/+ mice (total = 76 trials, n = 8) and 86.41% of ASIC3−/− mice (total = 104 trials, n = 15) chose the safe zone. Trials were not counted if the tested mouse did not choose a zone in 5 minutes. There was no significant difference between Asic3+/+ and Asic3−/− in choosing safe zone (p = 0.8665; no significant). Chi-square test was used to compare the data between genotypes.
(0.35 MB TIF)

Figure S3 Olfactory habituation. (A) An illustrated protocol to test olfactory habituation. ITI, inter-trial interval. (B) Asic3+/+ mice highly investigated the eppendorff in the first two trials and significantly decreased in investigation duration in trials 3 & 4. When a novel scent presented, mice increased in investigating on the novel scent. Asic3−/− mice showed similar pattern with Asic3+/+ Mice. There was no significant difference between the genotypes in each trial. *P<0.05, comparison between first trial and other trials. Data are presented as mean±s.e.m (each n = 9). ANOVA with post hoc test LSD was used to compare the difference between trials or genotypes.
(0.11 MB TIF)

Figure S4 Anxiety behavior and locomotion. Open-field test showed there were no differences among genotypes in the (A) times of entering center area, (B) percentage of time spending in center area/total time, (C) latency to entering center area, (D) distance traveled in center area. (E) There were no differences in total traveling distance in each time bins between genotypes. Asic3+/+, n = 8; Asic3+/−, n = 8; Asic3−/−, n = 7. ANOVA with post hoc test LSD was used to compare the difference among genotypes.
(0.19 MB TIF)

Figure S5 Comparison of pup retrieval activities on the first and second day. (A) The investigation latency showed no difference between the first and second day in each genotypes. (B) Both Asic3+/+ and Asic3+/− mice showed shorter retrieval latency for the 3rd on the second day than on the first day. However, in both Asic3−/− groups, retrieval latency for all three pups was not different between the first and second day (n = 9 Asic3+/+; n = 8 Asic3+/−; n = 8 Asic3−/−(he); n = 9 Asic3−/−(ko)). ANOVA with post hoc test LSD was used to compare the difference between days or genotypes. **P<0.01, comparison between Day1 and Day2. Data are mean±SEM.
(0.19 MB TIF)

Figure S6 Pup injury in pup retrieval tests. (A) During 30-min retrieval test, virgin Asic3−/− mice injured pups on their heads (middle left), feet (middle right) and bodies (bottom left). Moreover, Asic3−/− mice also caused infanticide (bottom right). Virgin Asic3+/+ (top left) and Asic3+/− (top right) mice did not cause injury to pups. (B) Percentage difference among genotypes in pup injury by mothers. Asic3+/+ mice, n = 9; Asic3+/− mice, n = 8; Asic3−/−(he) mice, n = 8; Asic3−/−(ko) mice, n = 9.
(0.43 MB TIF)

Figure S7 Age-dependent effect on maternal behaviors. Asic3+/− and Asic3−/− (combined Asic3−/−(he) and Asic3−/−(ko)) virgin mice were divided into two age groups, which were 8–12 and 12–16 weeks old. (A) Pup retrieval latency for each pup on day-1 trials. There was a significant difference between ages in 3rd pup retrieval latency in Asic3+/− mice (P = 0.0202). In Asic3−/− mice, the retrieval latency of 2nd (P = 0.011) and 3rd (P = 0.0181) pup was also different. (B) Retrieval latency for each pup on day-2 trials. (C) Investigation latency for the first pup on day-1 trials. (D) Investigation latency for the first pup on day-2 trials. (E) Ratios of time spent on crouching behaviors after all three pups were retrieved on the second day. (F) Duration of licking/grooming/sniffing behavior on the second day. The time spent on licking/grooming/sniffing was lower in 12–16 weeks of age than in 8–12 weeks of age in Asic3−/− mice (P = 0.03). Asic3+/− (8–12 weeks) n = 4; Asic3+/− (12–16 weeks) n = 4; Asic3−/− (8–12 weeks) n = 11; Asic3−/− (12–16 weeks) n = 6. Mann-Whitney U-test was used to compare the difference between ages. *P<0.05, comparison between 8–12 weeks and 12–16 weeks at Asic3+/− mice. #P<0.05, comparison between 8–12 weeks and 12–16 weeks at Asic3−/− mice. Data are mean±SEM.
(0.18 MB TIF)

Figure S8 Pup retrieval behaviors of mouse mothers. First, the nest area was identified after the offspring was born. Then the pups were separated from their mothers for 10 minutes and kept on a heating pad. After 10 minutes, five pups were put in the opposite side of nest area. The latency to retrieve pups to nest area was observed for 20 minutes. Asic3+/+ dams retrieved all pups to correct nest area quickly. In contrast, Asic3−/− dams often left pups in the original place or retrieved them to incorrect area.
(1.13 MB TIF)

Figure S9 Litter sizes at weaning. The numbers of pups in weaned ages were recorded from individual breeding pair. The dams was separated into three age groups, which were 8–12, 12–16, >16 weeks old. Generally, Asic3−/− dams showed less weaned pups than Asic3+/+ and Asic3+/− dams. At 8–12 weeks old, n = 25 Asic3+/+; n = 22 Asic3+/−; n = 18 Asic3−/−(he); n = 13 Asic3−/−(ko). At 12–16 weeks old, n = 39 Asic3+/+; n = 18 Asic3+/−; n = 17 Asic3−/−(he); n = 12 Asic3−/−(ko). At the ages >16 weeks, n = 77 Asic3+/+; n = 66 Asic3+/−; n = 30 Asic3−/−(he); n = 29 Asic3−/−(ko). ANOVA with post hoc test LSD was used to compare difference between genotypes. **P<0.01, comparison between Asic3+/+ and other genotypes. ##P<0.01, comparison between Asic3+/− and other genotypes. Data are mean±SEM.
(0.17 MB TIF)

Figure S10 Body weight at weaning. Body weights of pups were measured at weaning. No difference was found among genotypes in the average body weight during weaned ages (P>0.05, n = 968 Asic3+/+; n = 109 Asic3−/−(he); n = 523 Asic3−/−(ko)). This result indicated that the nutrient provided by Asic3−/− dams were normal as compared with Asic3+/+ dams.
(0.10 MB TIF)

Figure S11 Pups emitted USVs with longer bout duration when they confronted Asic3+/− or Asic3−/− virgin mice than when they confronted Asic3+/+ virgin mice. *P<0.05, **P<0.01, between Asic3+/+ and other genotypes. Bout number in Asic3+/+ group was 75; Asic3+/− group was 145; Asic3−/− group was 91.
(0.13 MB TIF)

Movie S1 Comparing the pup retrieval in Asic3+/+ and Asic3−/− dams. White circle indicated nest area. In the left, Asic3+/+ dam showed quickly retrieved all pups into nest area. In the right, Asic3−/− dam only retrieved one pup into nest area, but leaving others in the original place.
(8.81 MB MOV)

Movie S2 Virgin Asic3+/+ (upper panel) and Asic3−/−lower panel) mouse responded to pup USV in pup retrieval tests.
(2.35 MB MOV)

Text S1 Methods for Supporting Data
(0.03 MB DOC)
