# Supplementary figures and images for: Asic3−/− Female Mice with Hearing Deficit Affects Social Development of Pups
Source: PLoS One. 2009 Aug 4;4(8):e6508. doi: 10.1371/journal.pone.0006508 (PMC2714966; doi:10.1371/journal.pone.0006508)

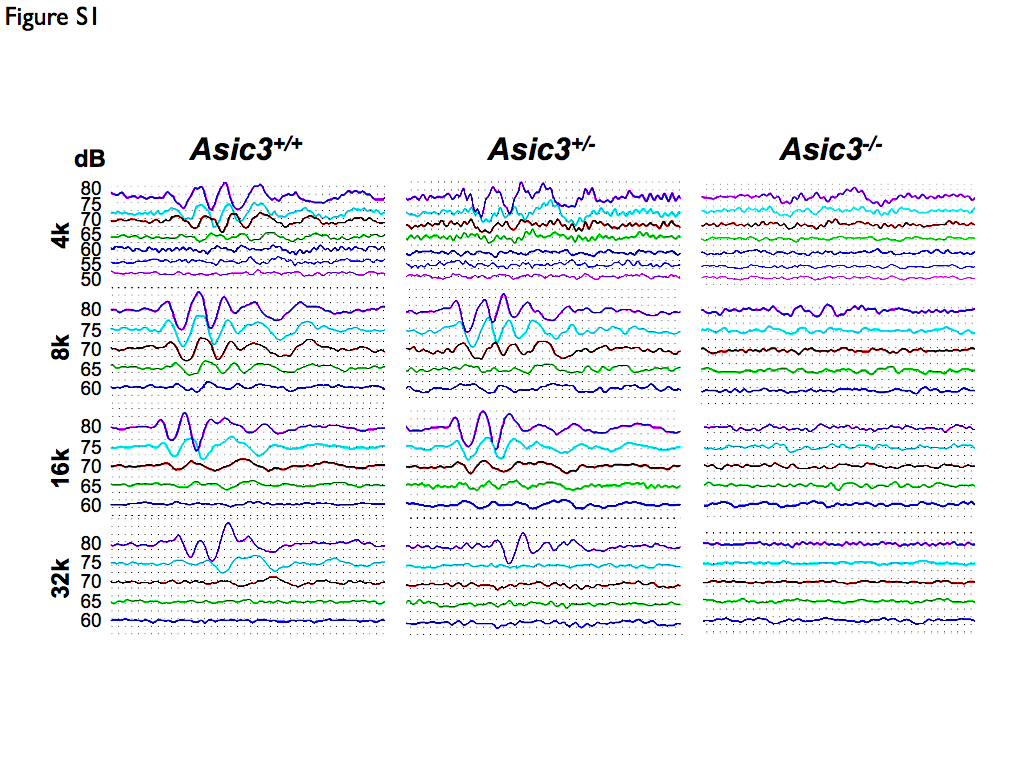

Supplement: Figure S1 — Representative 4-, 8-, 16- and 32-kHz tone burst-evoked auditory brainstem responses (ABRs) from mice at 12 weeks of age. Asic3−/− mice showed elevated hearing threshold with 4 and 8 kHz as compared with other genotypes. With 16- and 32-kHz tone bursts, Asic3−/− mice were nearly deaf, whereas other genotypes showed normal hearing. (0.40 MB TIF) [file pone.0006508.s001.tif]

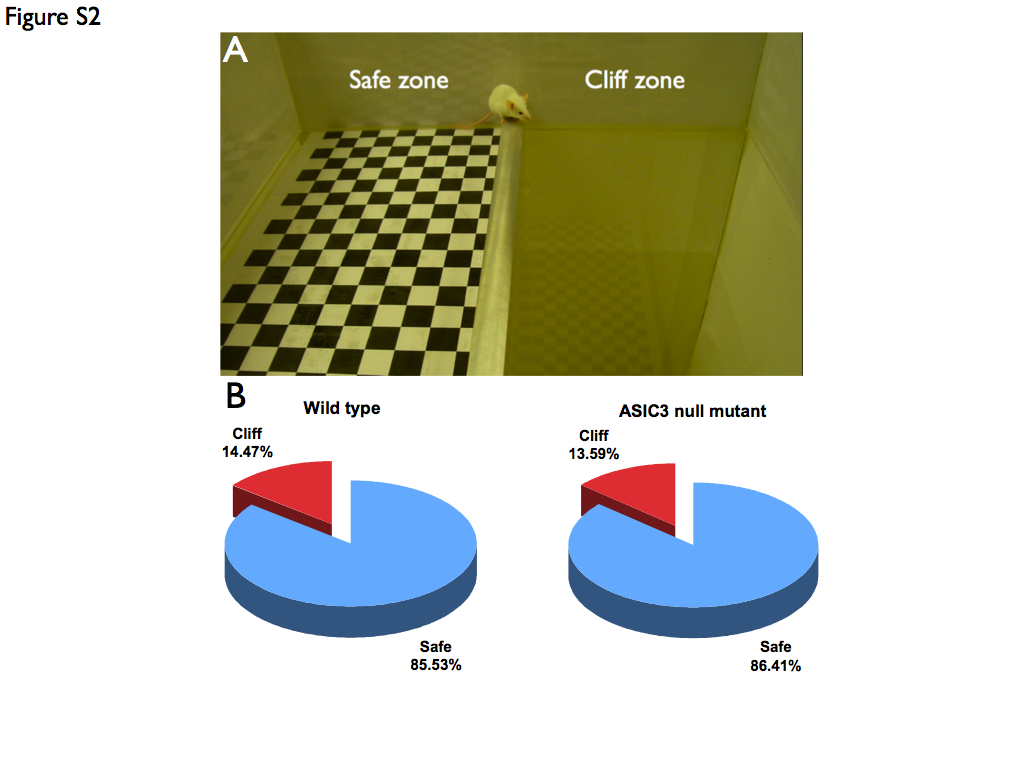

Supplement: Figure S2 — Visual cliff. (A) Apparatus: left side is the “safe” zone; right side is the “cliff” zone. Mice with normal vision will step down to the safe zone instead of cliff zone. Each mouse had 10 trials to choose the zone. The box were turned 180° after 5 trials to eliminate the memory effect. (B) 85.53% of ASIC3+/+ mice (total = 76 trials, n = 8) and 86.41% of ASIC3−/− mice (total = 104 trials, n = 15) chose the safe zone. Trials were not counted if the tested mouse did not choose a zone in 5 minutes. There was no significant difference between Asic3+/+ and Asic3−/− in choosing safe zone (p = 0.8665; no significant). Chi-square test was used to compare the data between genotypes. (0.35 MB TIF) [file pone.0006508.s002.tif]

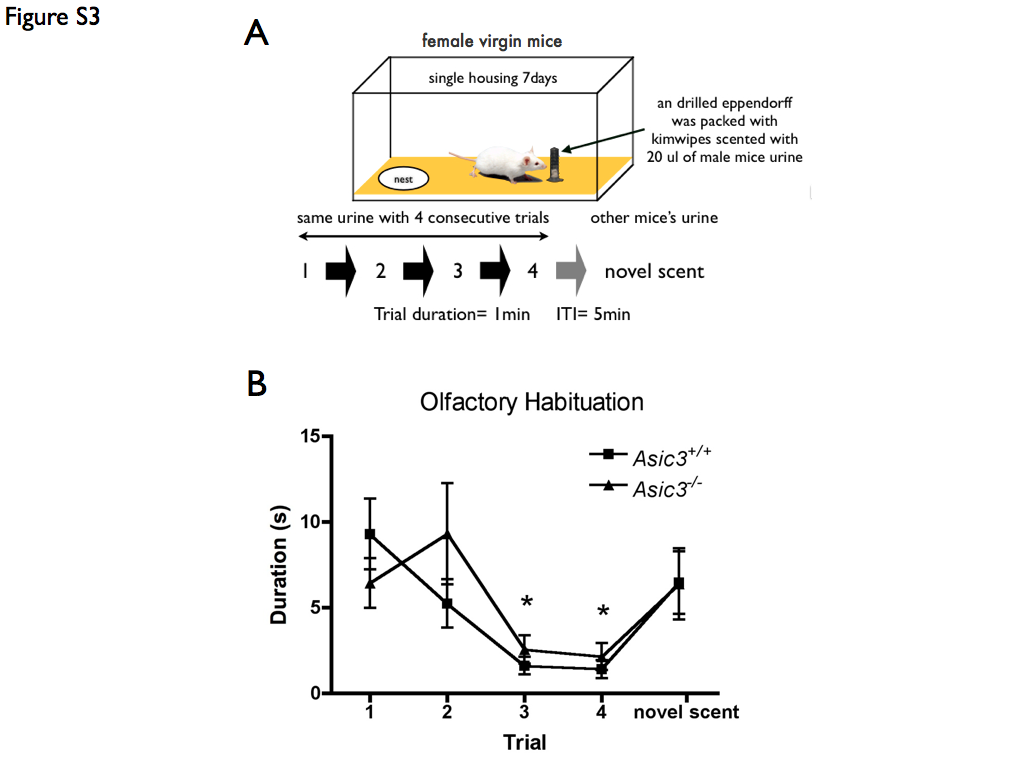

Supplement: Figure S3 — Olfactory habituation. (A) An illustrated protocol to test olfactory habituation. ITI, inter-trial interval. (B) Asic3+/+ mice highly investigated the eppendorff in the first two trials and significantly decreased in investigation duration in trials 3 & 4. When a novel scent presented, mice increased in investigating on the novel scent. Asic3−/− mice showed similar pattern with Asic3+/+ Mice. There was no significant difference between the genotypes in each trial. *P<0.05, comparison between first trial and other trials. Data are presented as mean±s.e.m (each n = 9). ANOVA with post hoc test LSD was used to compare the difference between trials or genotypes. (0.11 MB TIF) [file pone.0006508.s003.tif]

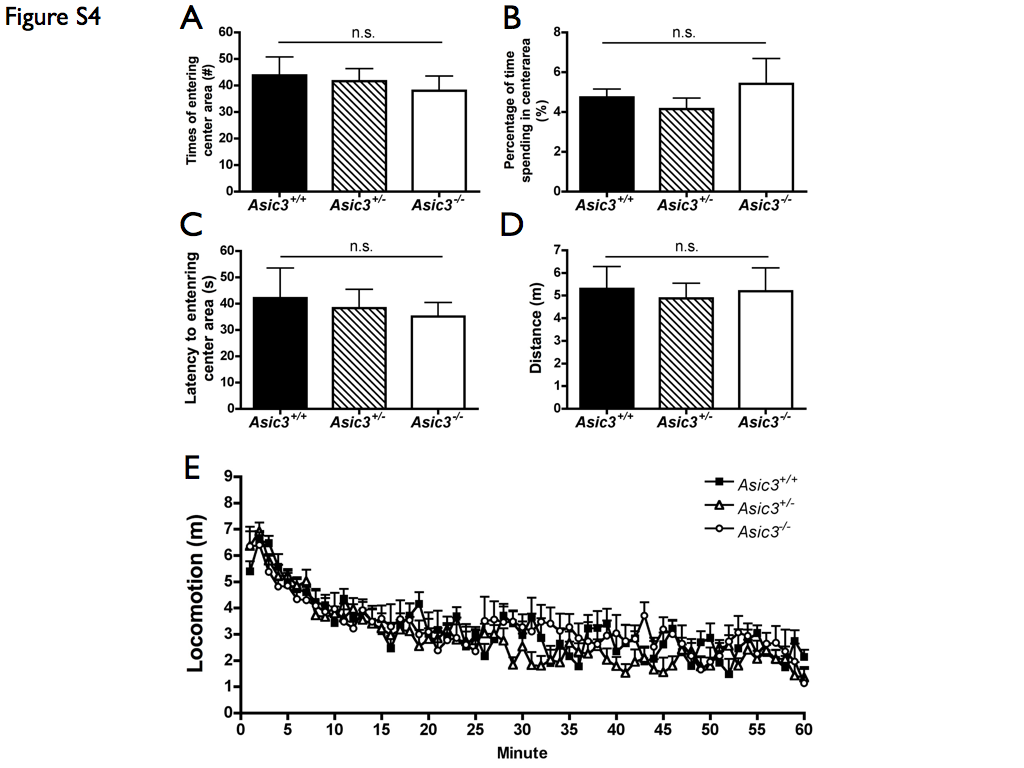

Supplement: Figure S4 — Anxiety behavior and locomotion. Open-field test showed there were no differences among genotypes in the (A) times of entering center area, (B) percentage of time spending in center area/total time, (C) latency to entering center area, (D) distance traveled in center area. (E) There were no differences in total traveling distance in each time bins between genotypes. Asic3+/+, n = 8; Asic3+/−, n = 8; Asic3−/−, n = 7. ANOVA with post hoc test LSD was used to compare the difference among genotypes. (0.19 MB TIF) [file pone.0006508.s004.tif]

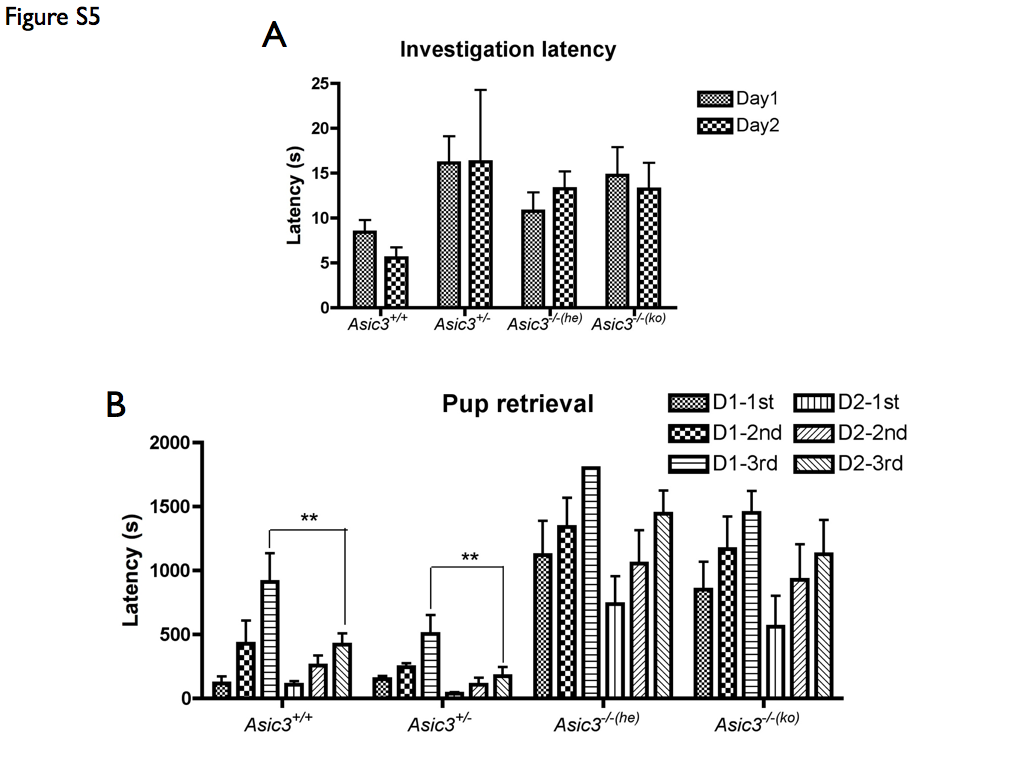

Supplement: Figure S5 — Comparison of pup retrieval activities on the first and second day. (A) The investigation latency showed no difference between the first and second day in each genotypes. (B) Both Asic3+/+ and Asic3+/− mice showed shorter retrieval latency for the 3rd on the second day than on the first day. However, in both Asic3−/− groups, retrieval latency for all three pups was not different between the first and second day (n = 9 Asic3+/+; n = 8 Asic3+/−; n = 8 Asic3−/−(he); n = 9 Asic3−/−(ko)). ANOVA with post hoc test LSD was used to compare the difference between days or genotypes. **P<0.01, comparison between Day1 and Day2. Data are mean±SEM. (0.19 MB TIF) [file pone.0006508.s005.tif]

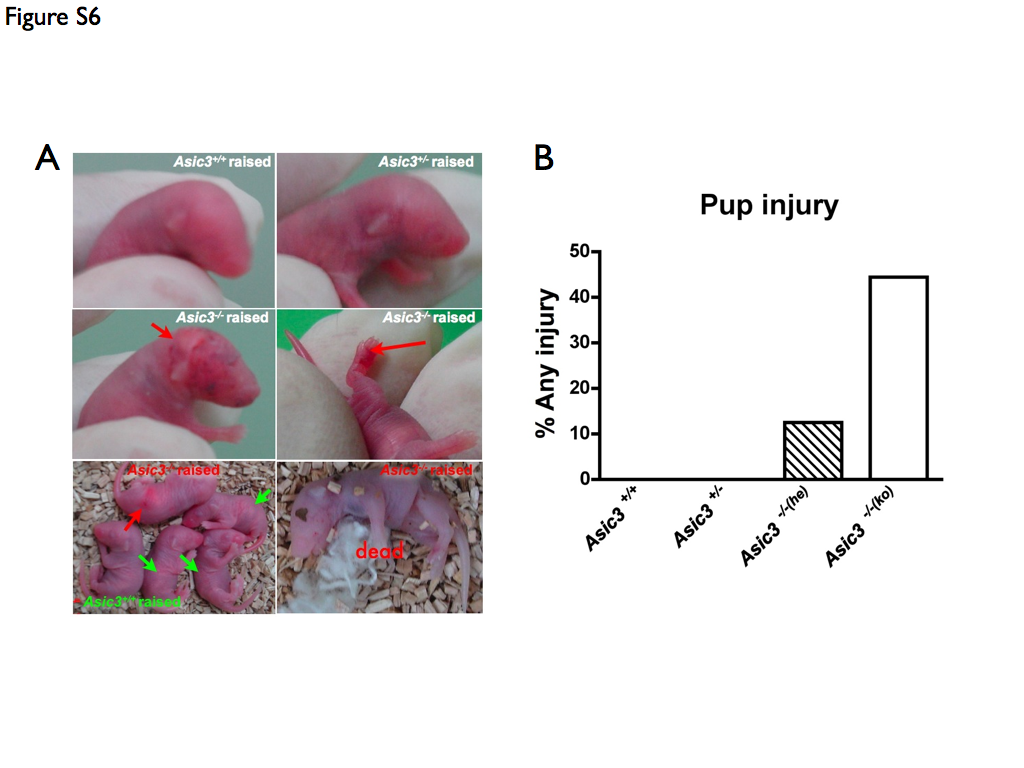

Supplement: Figure S6 — Pup injury in pup retrieval tests. (A) During 30-min retrieval test, virgin Asic3−/− mice injured pups on their heads (middle left), feet (middle right) and bodies (bottom left). Moreover, Asic3−/− mice also caused infanticide (bottom right). Virgin Asic3+/+ (top left) and Asic3+/− (top right) mice did not cause injury to pups. (B) Percentage difference among genotypes in pup injury by mothers. Asic3+/+ mice, n = 9; Asic3+/− mice, n = 8; Asic3−/−(he) mice, n = 8; Asic3−/−(ko) mice, n = 9. (0.43 MB TIF) [file pone.0006508.s006.tif]

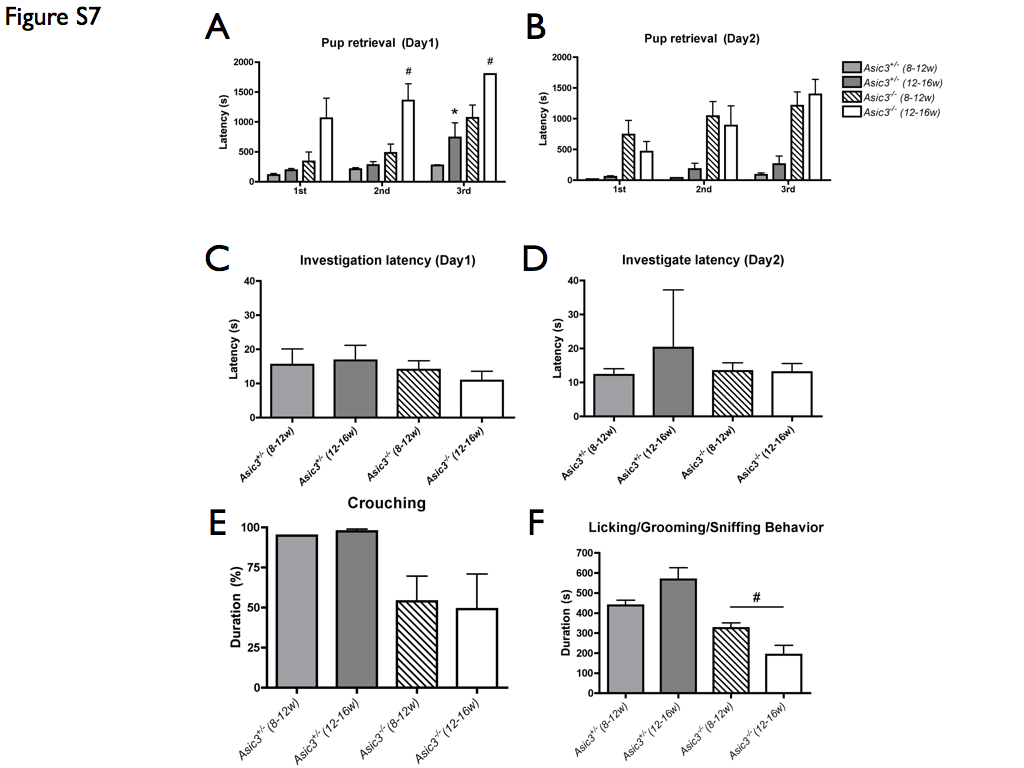

Supplement: Figure S7 — Age-dependent effect on maternal behaviors. Asic3+/− and Asic3−/− (combined Asic3−/−(he) and Asic3−/−(ko)) virgin mice were divided into two age groups, which were 8–12 and 12–16 weeks old. (A) Pup retrieval latency for each pup on day-1 trials. There was a significant difference between ages in 3rd pup retrieval latency in Asic3+/− mice (P = 0.0202). In Asic3−/− mice, the retrieval latency of 2nd (P = 0.011) and 3rd (P = 0.0181) pup was also different. (B) Retrieval latency for each pup on day-2 trials. (C) Investigation latency for the first pup on day-1 trials. (D) Investigation latency for the first pup on day-2 trials. (E) Ratios of time spent on crouching behaviors after all three pups were retrieved on the second day. (F) Duration of licking/grooming/sniffing behavior on the second day. The time spent on licking/grooming/sniffing was lower in 12–16 weeks of age than in 8–12 weeks of age in Asic3−/− mice (P = 0.03). Asic3+/− (8–12 weeks) n = 4; Asic3+/− (12–16 weeks) n = 4; Asic3−/− (8–12 weeks) n = 11; Asic3−/− (12–16 weeks) n = 6. Mann-Whitney U-test was used to compare the difference between ages. *P<0.05, comparison between 8–12 weeks and 12–16 weeks at Asic3+/− mice. #P<0.05, comparison between 8–12 weeks and 12–16 weeks at Asic3−/− mice. Data are mean±SEM. (0.18 MB TIF) [file pone.0006508.s007.tif]

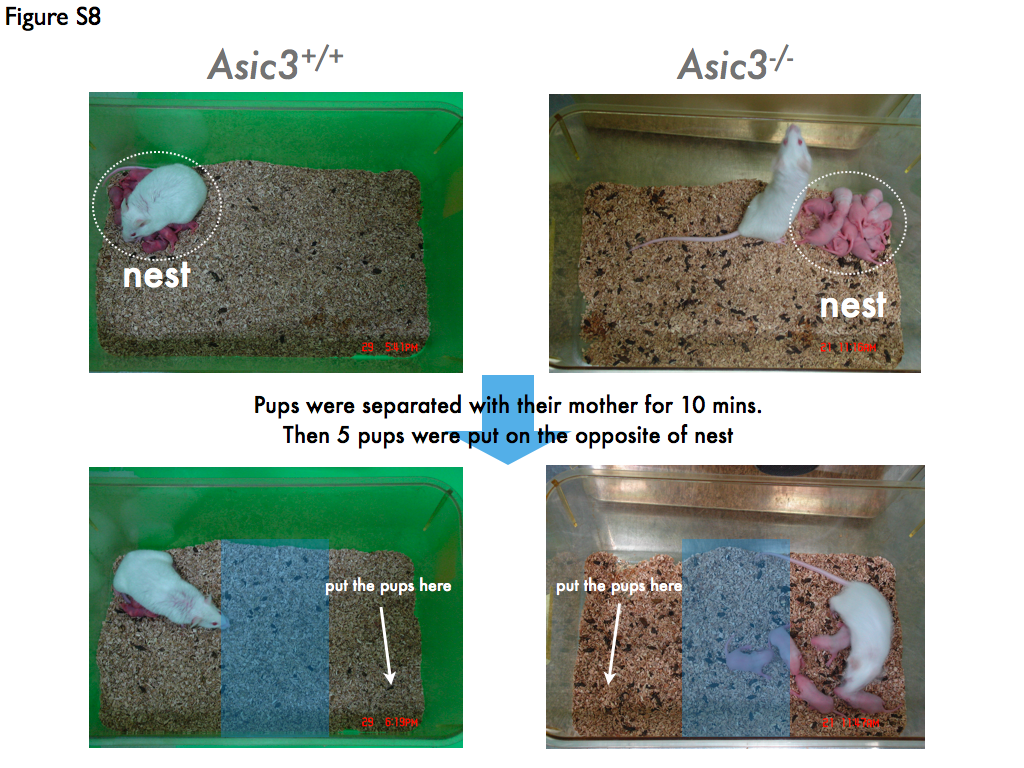

Supplement: Figure S8 — Pup retrieval behaviors of mouse mothers. First, the nest area was identified after the offspring was born. Then the pups were separated from their mothers for 10 minutes and kept on a heating pad. After 10 minutes, five pups were put in the opposite side of nest area. The latency to retrieve pups to nest area was observed for 20 minutes. Asic3+/+ dams retrieved all pups to correct nest area quickly. In contrast, Asic3−/− dams often left pups in the original place or retrieved them to incorrect area. (1.13 MB TIF) [file pone.0006508.s008.tif]

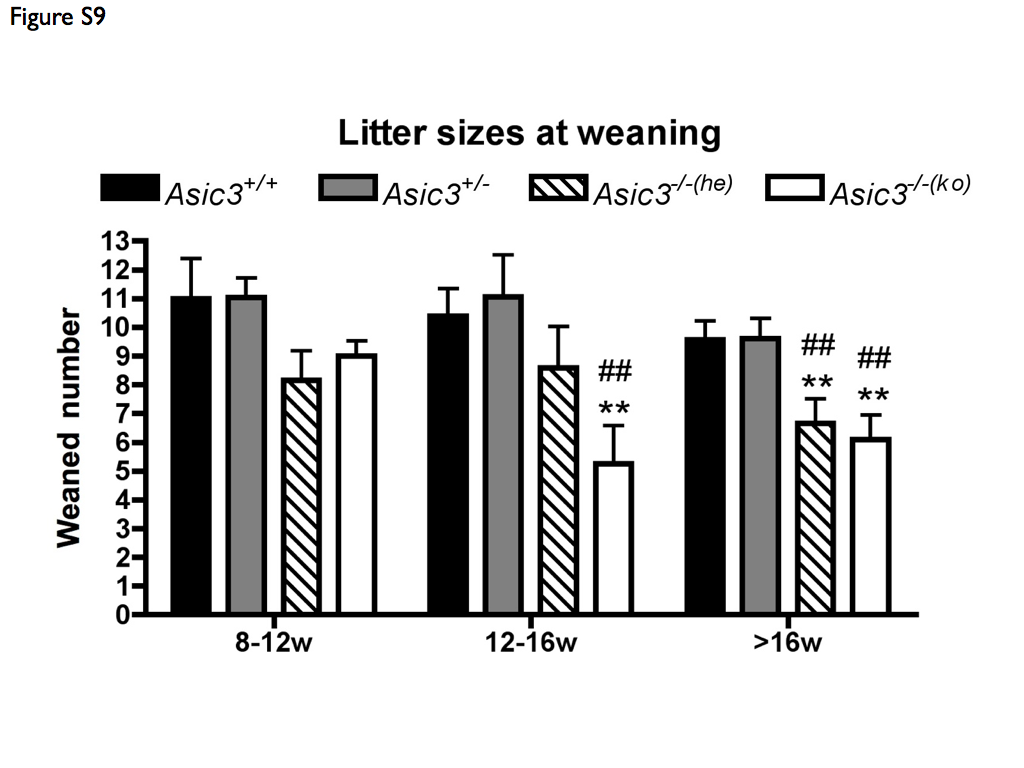

Supplement: Figure S9 — Litter sizes at weaning. The numbers of pups in weaned ages were recorded from individual breeding pair. The dams was separated into three age groups, which were 8–12, 12–16, >16 weeks old. Generally, Asic3−/− dams showed less weaned pups than Asic3+/+ and Asic3+/− dams. At 8–12 weeks old, n = 25 Asic3+/+; n = 22 Asic3+/−; n = 18 Asic3−/−(he); n = 13 Asic3−/−(ko). At 12–16 weeks old, n = 39 Asic3+/+; n = 18 Asic3+/−; n = 17 Asic3−/−(he); n = 12 Asic3−/−(ko). At the ages >16 weeks, n = 77 Asic3+/+; n = 66 Asic3+/−; n = 30 Asic3−/−(he); n = 29 Asic3−/−(ko). ANOVA with post hoc test LSD was used to compare difference between genotypes. **P<0.01, comparison between Asic3+/+ and other genotypes. ##P<0.01, comparison between Asic3+/− and other genotypes. Data are mean±SEM. (0.17 MB TIF) [file pone.0006508.s009.tif]

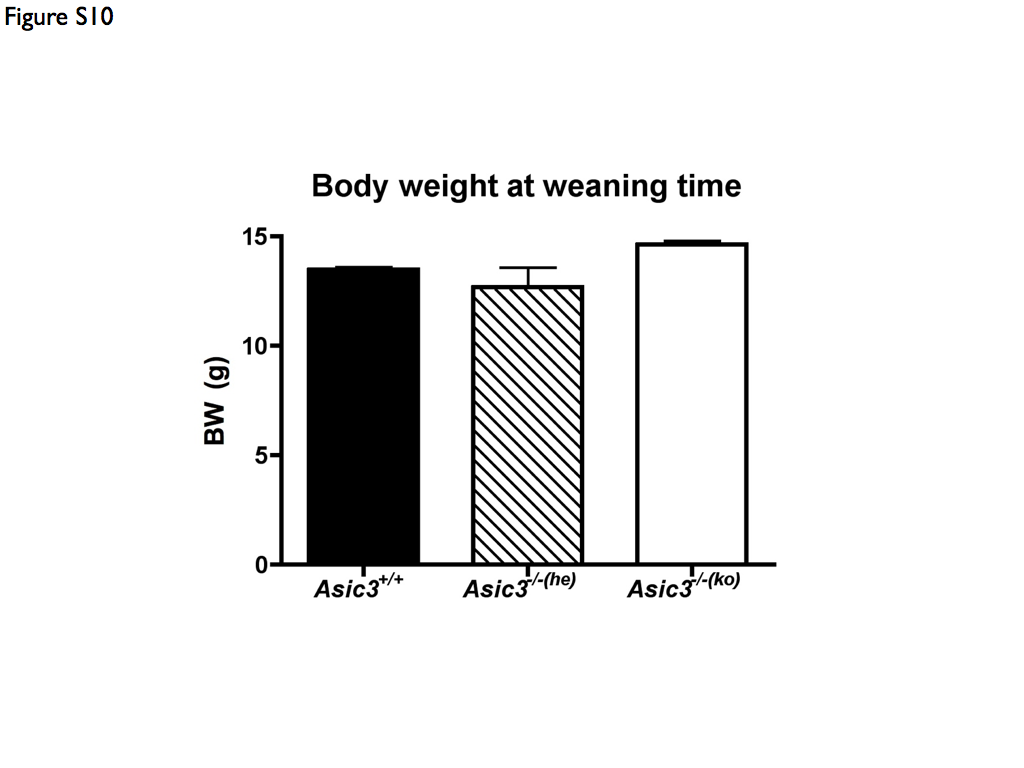

Supplement: Figure S10 — Body weight at weaning. Body weights of pups were measured at weaning. No difference was found among genotypes in the average body weight during weaned ages (P>0.05, n = 968 Asic3+/+; n = 109 Asic3−/−(he); n = 523 Asic3−/−(ko)). This result indicated that the nutrient provided by Asic3−/− dams were normal as compared with Asic3+/+ dams. (0.10 MB TIF) [file pone.0006508.s010.tif]

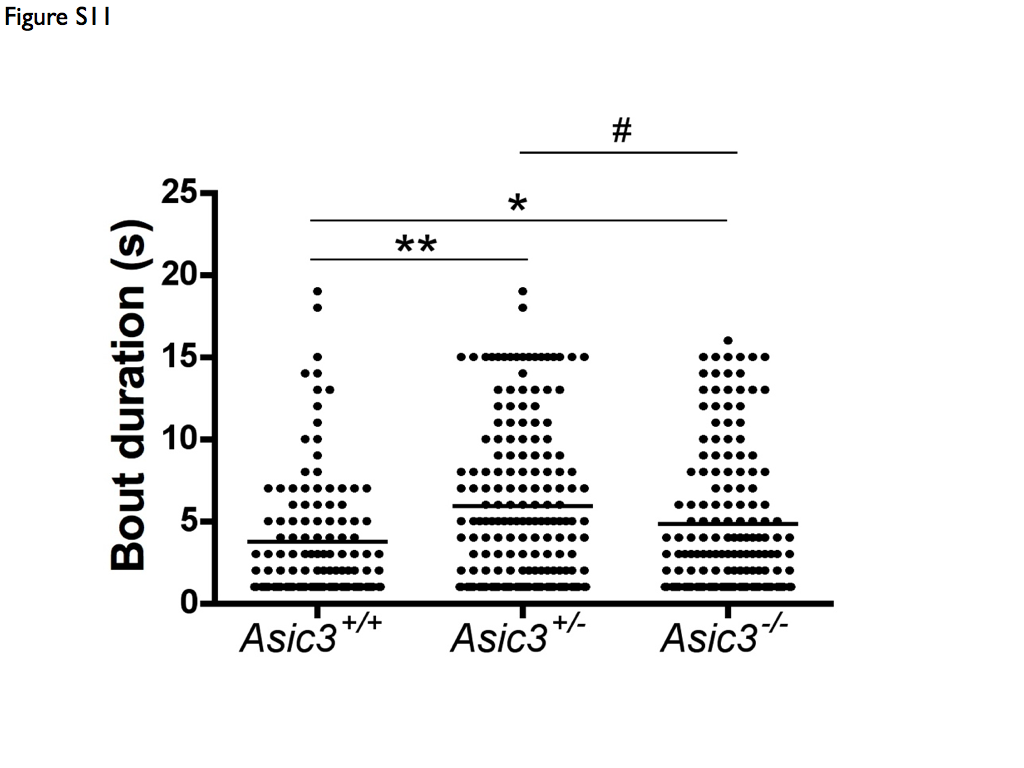

Supplement: Figure S11 — Pups emitted USVs with longer bout duration when they confronted Asic3+/− or Asic3−/− virgin mice than when they confronted Asic3+/+ virgin mice. *P<0.05, **P<0.01, between Asic3+/+ and other genotypes. Bout number in Asic3+/+ group was 75; Asic3+/− group was 145; Asic3−/− group was 91. (0.13 MB TIF) [file pone.0006508.s011.tif]
