# Supplementary material for: Asic3−/− Female Mice with Hearing Deficit Affects Social Development of Pups
Source: PLoS One. 2009 Aug 4;4(8):e6508. doi: 10.1371/journal.pone.0006508 (PMC2714966; doi:10.1371/journal.pone.0006508)
Supplement: Text S1 — Methods for Supporting Data (0.03 MB DOC) [file pone.0006508.s014.doc]

**Visual cliff**

The apparatus contains a transparent plastic box (62 x 62 x62 cm) with the four edges emerging 19 cm above the top and a metal rod running along the middle. The metal rod is 3.75 cm in height, 60 cm in length and 2.5 cm in width. The metal rod separates the box into two sections, one has a checker paper placed on the top surface of the box, whereas the other has the same checker paper placed on the bottom surface of the box. This experiment was run by placing the mouse on the metal rod and simply observing what decision the mouse made within 5 minutes, safe zone or cliff zone. Safe zone results represented the events of the mouse stepped down to the reasonable side, which has the checker paper on the top surface of the box. Cliff zone results represented the events of the mouse stepped down on the “fake cliff” side, which has the checker paper on the bottom surface of the box. A total of 22 mice were used in this study, including 8 wild type and 14 knockout mice. Each mouse was tested for 10 trials. To reduce the effect of learning and memory, the apparatus was turned 180 degrees after five trials so that the “safe zone” and the “cliff zone” were at different sides for the next five trials. The plastic surface had to be cleaned thoroughly between each trial in order to prevent mice from finding visual clues about depth. The percentages of choosing zones for each mouse were calculated. The difference between genotypes was compared by chi-square test.

**Olfactory habituation**

Animals were individually housed for at least 7 days before experiment. A drilled eppendorff containing kimwipes scented with 20 μl male mice urine was placed in the home cage for 1 min over 4 trials with an inter-trial interval (ITI) of five minutes. In the fifth dishabituation trial the original urine was replaced to another male mice urine. The time spent in olfactory investigation for each trial was recorded. Olfactory investigation was defined as direct nasal contact with the eppendorff. Mice with normal olfaction would decrease the investigation duration in trials 3 or 4, but increase when the novel scent presented. The urine investigation duration were compared between genotypes with unpaired student t-test.

**Open-field test**

The open field was a square area (48X48cm with 35cm height) constructed of clear Plexiglas. Mouse was placed in the arena, along the wall, and videorecorded for 60 min. All behaviors were recorded by CCD-computer and saved as .MPG format. The behaviors in the open field test were then analyzed by TopScan software (Clever System Inc., Reston, VA, USA). The center zone (16X16 cm) was defined at the middle of the open-field chamber. The numbers of entry, the duration of staying, the latency to cross into, and the distance traveled in the central zone were analyzed. The following behaviors were also recorded: grooming, rearing, slow speed movement and total distance traveled. The distance traveled in the whole area was measured to be the general locomotion activity.

**Maternal behavior of dams**

All females were housed individually once pregnant. Births were recorded in each morning. Each new dam was first observed for 5 min with minimal disturbance. All of her pups were then removed for 10-min isolation. Five pups were returned and placed at the opposite side from the nest in the dam’s cage or separate cages. Each mother was observed for 20 min with minimal disturbance. Retrieval was defined as the dam picking up a pup in her mouth and transporting it to the nest in wild-type mice. In the case of *Asic3^-/-^* dams, if she picked up and dropped the same pup more than once en route to the nest, the retrieval was not scored until the pup was in the original nest or placed in a position within the one third of the cage nearest the original nest. The retrieval test was repeated 4 times with the same 1- to 5-day-old pups.
